# Validation of an established deep learning auto-segmentation tool for cardiac substructures in 4D radiotherapy planning scans

**DOI:** 10.1016/j.phro.2022.07.003

**Published:** 2022-07-26

**Authors:** Gerard M. Walls, Valentina Giacometti, Aditya Apte, Maria Thor, Conor McCann, Gerard G. Hanna, John O'Connor, Joseph O. Deasy, Alan R. Hounsell, Karl T. Butterworth, Aidan J. Cole, Suneil Jain, Conor K. McGarry

**Affiliations:** a Cancer Centre Belfast City Hospital, Belfast Health & Social Care Trust, Lisburn Road, Belfast BT9 7AB, Northern Ireland; b Patrick G Johnston Centre for Cancer Research, Queen’s University Belfast, Lisburn Road, Belfast BT9 7AB, Northern Ireland; c Department of Medical Physics, Memorial Sloan Kettering Cancer Center, New York, NY, United States; d Department of Cardiology, Belfast Health & Social Care Trust, Lisburn Road, Belfast BT9 7AB, Northern Ireland

**Keywords:** Auto-segmentation, Deep learning, Cardiac substructures, Radiotherapy, Cardiotoxicity, Lung cancer

## Abstract

**Background:**

Emerging data suggest that dose-sparing several key cardiac regions is prognostically beneficial in lung cancer radiotherapy. The cardiac substructures are challenging to contour due to their complex geometry, poor soft tissue definition on computed tomography (CT) and cardiorespiratory motion artefact. A neural network was previously trained to generate the cardiac substructures using three-dimensional radiotherapy planning CT scans (3D-CT). In this study, the performance of that tool on the average intensity projection from four-dimensional (4D) CT scans (4D-AVE), now commonly used in lung radiotherapy, was evaluated.

**Materials and Methods:**

The 4D-AVE of n=20 patients completing radiotherapy for lung cancer 2015–2020 underwent manual and automated cardiac substructure segmentation. Manual and automated substructures were compared geometrically and dosimetrically. Two senior clinicians also qualitatively assessed the auto-segmentation tool’s output.

**Results:**

Geometric comparison of the automated and manual segmentations exhibited high levels of similarity across parameters, including volume difference (11.8% overall) and Dice similarity coefficient (0.85 overall), and were consistent with 3D-CT performance. Differences in mean (median 0.2 Gy, range −1.6–0.3 Gy) and maximum (median 0.4 Gy, range −2.2–0.9 Gy) doses to substructures were generally small. Nearly all structures (99.5 %) were deemed to be appropriate for clinical use without further editing.

**Conclusions:**

Cardiac substructure auto-segmentation using a deep learning-based tool trained on a 3D-CT dataset was feasible on the 4D-AVE scan, meaning this tool is suitable for use on 4D-CT radiotherapy planning scans. Application of this tool would increase the practicality of routine clinical cardiac substructure delineation, and enable further cardiac radiation effects research.

## Highlights

• Cardiotoxicity is a common complication of lung cancer radiotherapy.
• Segmentation of cardiac substructures is time-consuming and challenging.
• Deep learning segmentation tools can perform this task in 3D and 4D scans.
• Performance is high when assessed geometrically, dosimetrically and clinically.
• Auto-segmentation tools may accelerate clinical workflows and enable research.

## 1 Introduction

Segmentation of tumors and organs-at-risk (OARs) is a pivotal step in contemporary, inverse-planned radiotherapy (RT). Prospectively defining clinical dose goals for these structures, allows tumor control probability to be maximised while normal tissue complication probability is minimised [1], [2]. The delineation of structures on planning scans is both time-consuming [3] and prone to inter-operator variability [4], with the latter resulting in negative clinical impact [5]. Overcoming these drawbacks, automated segmentation has been an interdisciplinary research focus, with tools now available for several primary tumors [6] and associated regional OARs [7], [8].

Incidental cardiac radiation dose correlates negatively with survival in lung cancer [9], [10], [11], [12] and particular regions, such as the heart base [12], [13], [14], may mediate this effect. Moreover, dose-volume characteristics for several of the specific cardiac substructures are important for predicting toxicity after conventional fractionation lung cancer RT [15], [16], [17], [18], [19], [20]. The cardiac substructures are challenging to contour on RT planning computed tomography (CT), due to geometry complexity, limited intracardiac soft tissue definition and cardiorespiratory motion artefact [21]. The imminent requirement to incorporate substructures in treatment planning is an opportunity to embed a cardiac segmentation tool into RT workflows.

To this end, in previous studies, a neural network was trained from 240 lung cancer treatment plans [22] to generate a whole heart and pericardium structure, and 10 cardiac substructures, based on the most established cardiac atlas [23]. This tool delineates the structures on standard free-breathing three-dimensional planning CT scans (3D-CT) with a high performance in 10 s, regardless of whether there is contrast enhancement [22]. The substructures delineated include the four cardiac chambers and four great vessels. When applied to the RTOG-0617 cohort, the whole heart algorithm from this tool was shown to represent the cardiac dosimetry more accurately than the original manual contours used in the trial [24].

For improved motion management, the use of four-dimensional (4D) planning scans (4D-CT) has largely replaced 3D-CT planning in contemporary lung cancer RT [25]. Validation of the deep learning auto-segmentation tool in the 4D setting is therefore required for its clinical implementation. In this study, we hypothesised that the performance of the tool in the average intensity projection of 4D-CT scans (4D-AVE) would be comparable to 3D-CT using geometry, volume, dosimetry and clinical acceptability metrics.

## 2 Materials & methods

### 2.1 Study design

The 4D-AVE dataset for 20 patients that completed radical RT for lung cancer between 2015 and 2020 at a single centre were selected based on a random number generator for this retrospective study. RT treatment plans underwent manual and automated cardiac segmentation for comparison of the deep learning-based tool’s performance. This modest sample size was prospectively chosen given the resemblance of the research to a feasibility study, and due to the labour-intense nature of cardiac contouring. There was no stratification by presence/absence of contrast as the algorithm was initially trained using both scenarios (46 % contrast; 54 % non-contrast). Governance was granted and ethical approval was waived by the Belfast Health & Social Care Trust (IRAS ID 293181) and the study was sponsored by Queen’s University Belfast.

### 2.2 Radiotherapy treatment plans

Scans of 20 patients who underwent 4D-CT planning were included. Patients were scanned in the supine position, immobilised using a knee rest and thorax board, with arms holding a T-bar above their head. Scans were performed during quiet respiratory motion using the Varian RPM system (Varian Medical Systems, Palo Alto, CA, USA) with the GE Advantage Sim 4D application (GE Medical Systems, Milwaukee, WI, USA). Ten phase bins were created to generate the 4D-AVE used for this study. CT images at 2.5 mm slice width were acquired from the cricoid to the second lumbar vertebra with intravenous contrast when clinically appropriate. Volumetric modulated arc therapy (VMAT) treatment plans were calculated for all patients based on the phase-binned 4D-CT using the Varian AAA 13.6.23 algorithm on the Varian Eclipse treatment planning system.

### 2.3 Manual segmentation

Eight cardiac substructures were manually delineated as the gold standard for comparison, using the 4D-AVE for all patients using standard mediastinal window-level settings, or alternatives when necessary. The right atrium (RA), left atrium (LA), right ventricle (RV), left ventricle (LV), aorta (AO), pulmonary artery (PA), superior vena cava (SVC) and inferior vena cava (IVC) were contoured on Eclipse. A whole heart (WH) structure was also segmented, with the superior border defined as the most inferior CT slice showing the pulmonary trunk bifurcating into the left and right pulmonary arteries. A ‘pericardium’ (PC) structure was generated by duplicating the WH structure and extending this to the superior extent of the aortic arch. Composite bilateral atria and ventricle structures were generated using the union function. All manual delineations were completed by the same clinical oncologist (GW) using the Feng atlas [23] and were then verified, and modified if required, by a senior thoracic radiation oncologist (GH) and a senior cardiologist (CMC) simultaneously.

### 2.4 Auto-Segmentation

The 4D-AVE scans were imported to MATLAB version 2020b (The Mathworks, Inc., Massacusetts, USA), where the validated deep learning segmentation tool [22] was applied. The deep neural network architecture was previously trained for label prediction of all pixels on individual CT slices as either within one of the cardiac structures, or the background image. Initially the anonymised CT data and structure (lungs only) files were uploaded within the Computational Environment for Radiotherapy Research (CERR) platform [26]. The CT image is cropped by the algorithm, using the medial walls and craniocaudal extent of the lung structures as limits on the region from which to begin generating structures. As part of the algorithm’s label prediction process, the input data undergoes random cropping, horizontal and vertical flipping, and rotation by 10°, as the segmentations are created. Once complete, the finalised individual slices are then stacked back together to generate a 3D segmentation. The time taken for the algorithm to complete is dependent on the computing hardware available, ranging from seconds only [22] to approximately 10 minutes in our study.

Automated structures were then imported back into Eclipse. Post-processing steps were applied to all structures, limited to removing structures < 0.5 cc (great vessels) or < 1.0 cc (all others) and the filling of cavities < 2 cc. Manual contours of the descending AO and IVC were cropped to the most inferior slice of the corresponding auto-contour, in order to pragmatically guarantee robust comparison of these structures, given that there are no published atlases for these substructures at the time of writing. Prior to geometric and dosimetric analysis, structures were amended on slices where a boundary was deemed to be suboptimal, as per section 2.6, with original versions retained for assessment of the impact of any changes.

### 2.5 Quantitative evaluation

Manual and automated structures were geometrically compared by percentage volume difference (VD), centroid shift (CS), Dice similarity coefficient (DSC), and 95 % Percentile Hausdorff distance (HD95) using Slicer-RT (PerkLab, Ontario, Canada) [27]. To evaluate dosimetric impact, the mean dose and maximum dose to 0.5 cc (Dmax) of the automated structures as calculated in Eclipse were compared against the manual contours.

### 2.6 Qualitative evaluation

A senior cardiologist (CMC) and senior thoracic radiation oncologist (GH) assessed the output of the auto-segmentation tool output structures following post-processing, prior to minor amendments, according to the scale used in the deep learning tool’s original publication [22]. Based on their theoretical suitability for treatment planning including substructure dose constraints, individual structures were rated as ‘good’, ‘acceptable’, ‘in need of amendment’ (NOA) or ‘poor’, according to the number of slices requiring amendment (see Supplementary Table 1). Both ‘good’ and ‘acceptable’ ratings equate to a whole structure being of sufficient quality without further modification. The maximum number of slices that required modification to achieve a perfect delineation was also recorded.

### 2.7 Statistical analysis

Following data collection, statistics were calculated using Prism v8.3.0 (GraphPad Software, San Diego, California, USA). Median, maximum and minimum values are displayed and were used for significance testing as the vast majority of data were not normally distributed according to the Shapiro-Wilk test. The significance of differences between parameters in manual and automated contours were tested using the Mann-Whitney U test, used for comparing non-parametric, unpaired data. Bland-Altman analyses were undertaken to assess bias, and Spearman’s r values were calculated to assess correlation. For DSC and HD95 data, comparison was made with summary statistics from the original publication [22] using Mann-Whitney tests, to allow benchmarking of the presented work.

## 3 Results

### 3.1 Patients

**Fig. 1:**
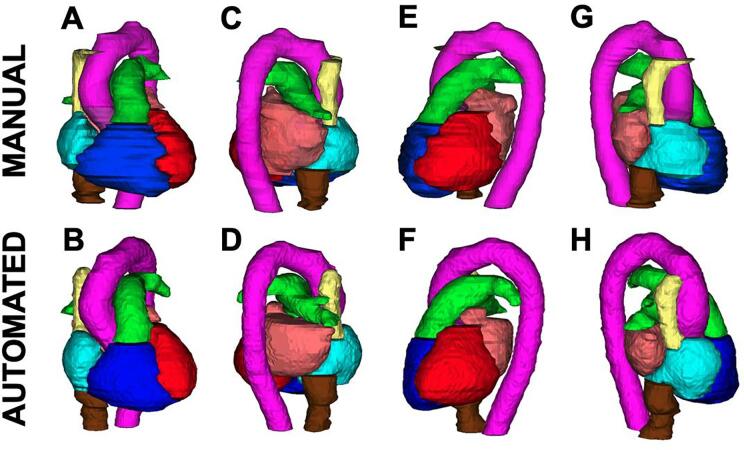
Three-dimensional reconstruction of cardiac substructures from a representative patient from the anterior (A, B), posterior (C, D), left (E, F) and right (G, H) perspectives, based on manual (top) and automated delineations (bottom) (right atrium = cyan; left atrium = orange; right ventricle = blue; left ventricle = red; pulmonary artery = green; aorta = magenta; superior vena cava = yellow; inferior vena cava = brown)

The median age of the cohort was 70.5 years, 7 of whom were female. Additional patient characteristics are summarised in Supplementary Table 2. Intravenous contrast was administered for 70 %, and all patients exhibited calcification which was mild, moderate or severe in 25 %, 45 % and 30 % respectively. A 3D reconstruction of the automated and manual contours for a representative patient are included in Fig. 1.

### 3.2 Comparison of geometry

**Fig. 2:**
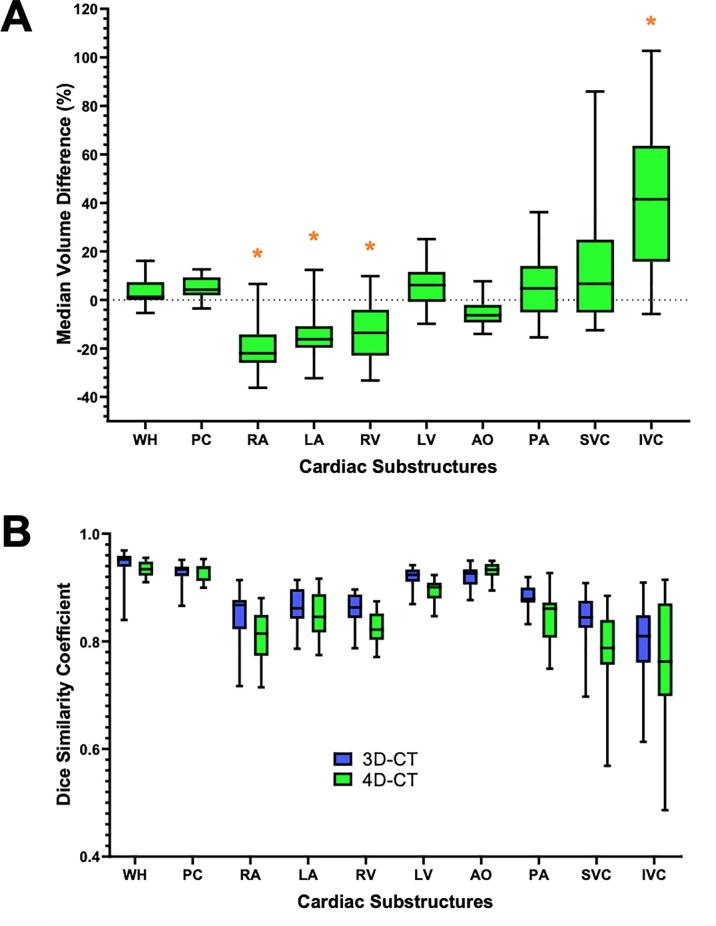
A) Box plot of percentage volume difference between the automated and manual delineations, relative to the manual contour. B) Box plot of DSCs for each substructure, comparing data from this manuscript with the original publication. (WH = whole heart; PC = pericardium; RA = right atrium; LA = left atrium; RV = right ventricle; LV = left ventricle; AO = aorta; PA = pulmonary artery; SVC = superior vena cava; IVC = inferior vena cava).

Automated volumes were smaller than manual for the chambers, except LV, and larger for the great vessels, except AO, as shown in Fig. 2A and Supplementary Table 3. The median VD for the WH was 1.3 % and the highest and lowest magnitude median VDs amongst the cardiac substructures were for the LV (6.1 %) and IVC (41.5 %) respectively. Statistically significant median VDs were found for RA, LA, RV and IVC. The overall median absolute VD across all substructures was 11.8 % (range 6.5–41.5). Comparing the volumes, there were low levels of bias as shown by good spread in the points, and few points out-with the limits of agreement for the majority of structures in Bland-Altman plots (see Supplementary Fig. 1). Values were also strongly correlated, with a mean Spearman’s r value of 0.90 across all cardiac structures (see Supplementary Table 4).

As shown in Fig. 2B and Supplementary Table 5, DSC values ranged 0.76–0.94 across the structures, with the worst performance on average for IVC at 0.76, and the best for PC at 0.94. The median DSC across all structures was 0.85 and the median DSC across patients was 0.82, 0.92 and 0.86 for minimum, maximum and median of respectively. In addition, there was good similarity in the DSCs with the original published cohort (p = 0.27).

Generally CS was approximately 3–4 mm for each structure, though was slightly better for AO at 1.7 mm and slightly worse for PA at 5.7 mm, as shown in Supplementary Table 6. Regarding the directionality of the X, Y and Z components of these shifts, there was a suggestion of a systematic effect in the X and Z axes, with negative values found for medians in 8/10 (ie right shifts) and 3/10 (ie inferior shifts) structures respectively. This was not observed in the Y axis (5/10 negative values) and moreover, as shown in Supplementary Table 7, the magnitude of the median shifts per structure were typically < 2 mm across substructures in all directions.

The HD95 values for the automated structures on 4D-AVE were generally small, with a median of 7.1 mm, and the substructures with the lowest and highest HD95s being the AO (2.4 mm) and the RV (8.9 mm). These data were clinically comparable to the published 3D-CT data, which had a median and range of 5.9 mm and 3.1–7.3 mm. Further details on the HD95 values are available in Supplementary Table 8.

### 3.3 Comparison of dose characteristics

**Fig. 3:**
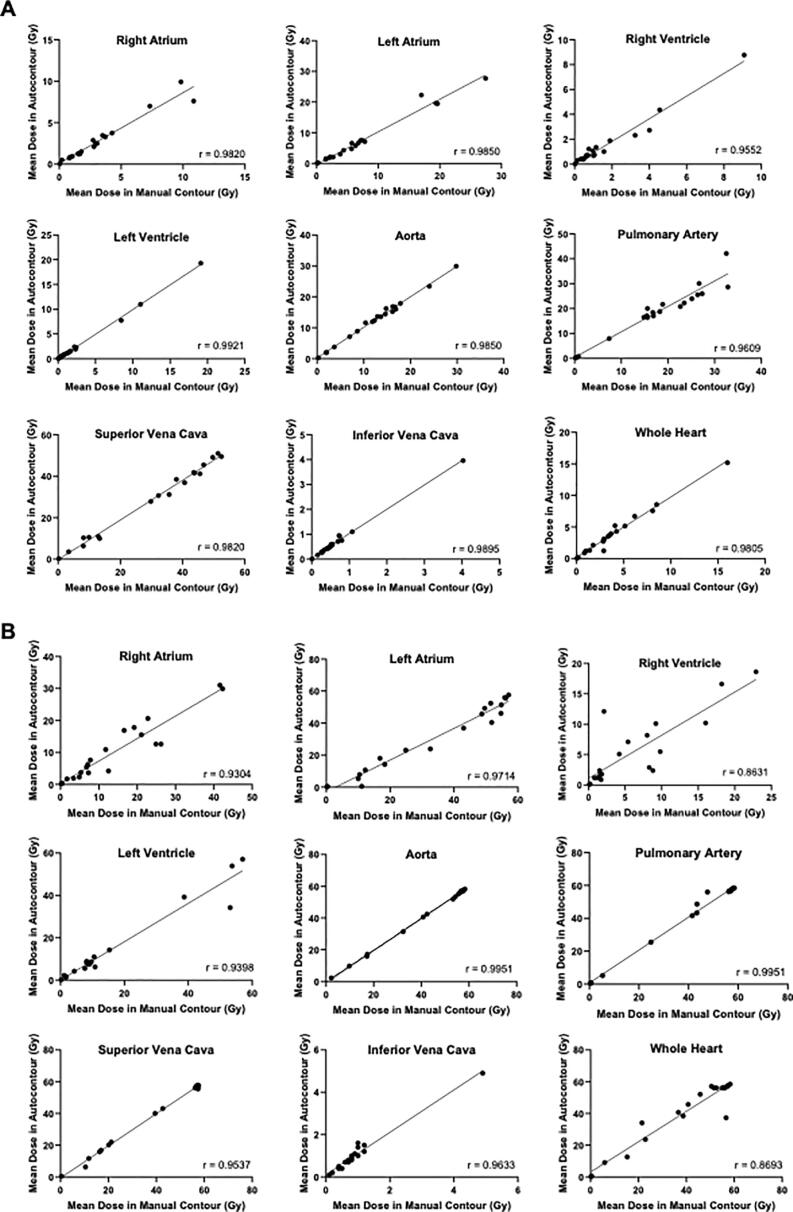
Scatter plots of mean (A) and maximum (B) doses (Gy) to automated and manual delineations, with Spearman correlation values displayed. (WH = whole heart; PC = pericardium; RA = right atrium; LA = left atrium; RV = right ventricle; LV = left ventricle; AO = aorta; PA = pulmonary artery; SVC = superior vena cava; IVC = inferior vena cava).

Mean and maximum doses to structures were not significantly different between the automated and manual segmentations on 4D-AVE scans, with a median absolute difference of 0.2 Gy (range 0–1.7 Gy) and 0.4 Gy (range 0.1–2.2 Gy) for mean dose and Dmax respectively. Differences were generally small for all cardiac structures, especially for mean dose, as shown in Supplementary Tables 9 and 10. Levels of correlation were high for both mean dose and Dmax, with median values of 0.99 and 0.96 respectively, as shown in Fig. 3. There were also low levels of bias, given the spread in the points in the Bland-Altman plots and low number of outlying points (see Supplementary Fig. 2, Fig. 3). The performance of the tool in each structure varied by parameter, but there was a pattern of lower dose differences for the RV and LV. Per patient across all structures, differences in mean dose were generally ± 2 Gy. Similarly for Dmax, differences were generally between −7 Gy and + 4 Gy across all structures per patient.

### 3.4 Qualitative comparison

**Fig. 4:**
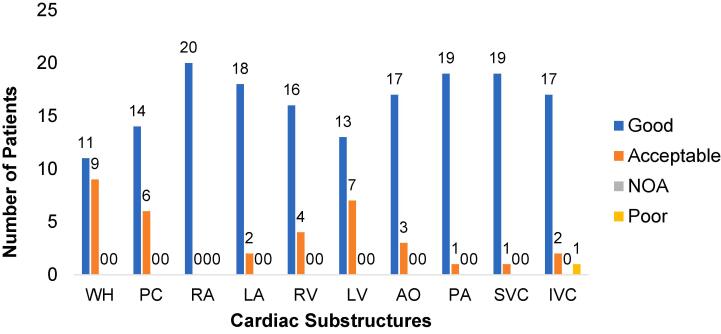
Qualitative evaluation of automated segmentation of 4D-CT RT planning scans for the whole heart and individual cardiac substructures for 20 patients with lung cancer.

**Fig. 5:**
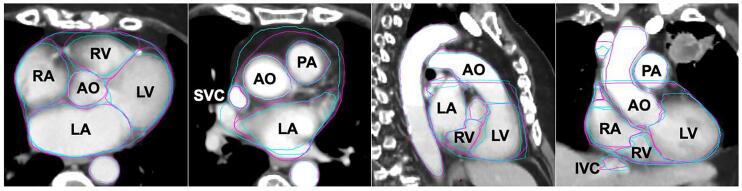
Representative cross-sectional images of the manual (purple) and automated (cyan) segmentations of the substructures on the average intensity projection scan, in the transverse (A–B), sagittal (C) and coronal (D) planes. (WH = whole heart; PC = pericardium; RA = right atrium; LA = left atrium; RV = right ventricle; LV = left ventricle; AO = aorta; PA = pulmonary artery; SVC = superior vena cava; IVC = inferior vena cava; NOA = in need of amendment). (For interpretation of the references to colour in this figure legend, the reader is referred to the web version of this article.)

Virtually all structures (99.5 %) and all patients (19 of 20) were deemed to be appropriate for clinical use without further editing by two senior clinicians (CMC, GH) according to criteria used by Haq et al, as shown in Fig. 4. Minor modifications of the automated contours (median of 2 slices for 18 % structures) led to insignificant changes in geometry only (see Supplementary Data File). Automated and manual delineations on the 4D-AVE scan are shown in Fig. 5.

## 4 Discussion

Cardiac substructure auto-segmentation has come to the fore in recent years owing to an increased interest in radiation effects on the heart [28], [29]. Recent studies have elicited dose–response relationships for several component structures [15], [16], [17], [18], [19], [20] and trials are underway to test the effect of sparing these regions [30]. As the application of artificial intelligence is explored in RT, volume delineation serves as a logical starting point [31], [32], [33]. By accurately performing complex and time-consuming delineation tasks, clinician availability for alternate activities could be increased and treatment planning delays reduced [34].

**Fig. 6:**
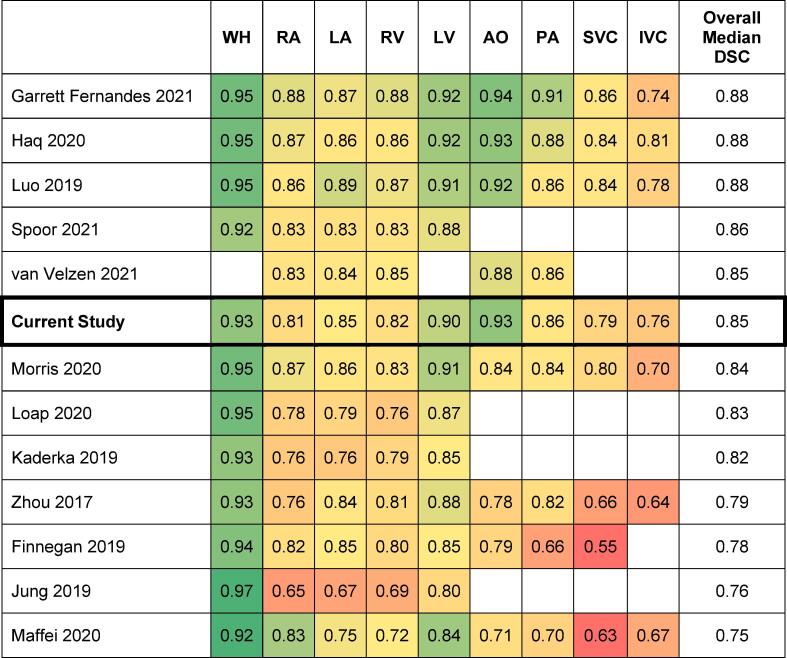
A heat map of DSCs among studies evaluating novel auto-segmentation tools for the cardiac substructures. (WH = whole heart; RA = right atrium; LA = left atrium; RV = right ventricle; LV = left ventricle; AO = aorta; PA = pulmonary artery; SVC = superior vena cava; IVC = inferior vena cava).

In this study, a deep learning-based cardiac substructure auto-segmentation tool developed for use in 3D-CT scans was retrospectively evaluated in 20 patients that underwent 4D-CT planning. This particular tool was selected from the literature [35], [36], [37], [38], [39], [40], [41], [42], [43], [44], [45], [46] due to its superiority in terms of structures included, performance metrics, applicability to lung cancer and availability. The cardiac substructure auto-segmentation available tools at the time of writing are compared in Supplementary Table 11. The median DSC across all substructures for the presented tool evaluated on 4D-CT scans compares well with other approaches, as shown in the heatmap below (Fig. 6). Of note, some papers from Supplementary Table 11 are not represented as none of the presented tool’s substructures were included, or because DSC were not reported. Furthermore, the selected articles were heterogenous in their calculation of summary statistics from individual cases, with some using mean and others using median.

In evaluating the geometric, dosimetric and clinical acceptability metrics of the deep learning tool on the 4D-AVE scans, the automated contours were found to be comparable with manual contours, with median DSC, centroid shift and mean dose difference of 0.85, 3.6 mm and 0.2 Gy. The overall performance of the tool per substructure varied according to parameter observed, for example there was a trend for lower performance on the RV (low DSC, high VD, high HD95) and higher performance on the PA (high DSC, small VD, low HD95).

Several of the structures with lower similarity between manual and auto-segmentation are challenging to delineate reproducibly as there is no atlas (e.g. for PA) or have an uncertain anatomical boundary on CT (e.g. for RA and LA), explaining the lower performance. The narrow inter-patient range in all of the parameter’s averages considering all substructures suggests that patients are contoured with similar degrees of ‘ease’ or ‘difficulty’. However, some structures had consistent differences in volume or dose across most patients (e.g. the RA had higher volumes and the SVC had lower mean doses). Interestingly, those structures with larger VDs did not appear to have larger dose differences.

Reassuringly overall, differences in the summary statistics between our 4D-CT data and the original 3D-CT publication were clinically insignificant, despite manual segmentations being completed by different operators [22]. Of note, the automated contours were found to be clinically acceptable overall, including those structures with larger VDs. Furthermore, clinician adjustment appeared to be unwarranted as geometric impact of resulting adjustments were shown to be negligible (see Supplementary Data File). The only anomaly with any frequency for the neural network was delineating the inferior WH and PC slices, where there is poor soft tissue contrast and considerable motion artefact at the interface with the diaphragm.

It is noteworthy that the presented auto-segmentation tool overcame the challenges of cardiac substructure delineation to produce contours equivalent to manual contours. The impediments include complex anatomy, frequent absence of contrast enhancement, poor soft tissue definition eg between the myocardium and pericardial fat, and biological variability eg vascular calcification or aneurysm. In addition, by using the 4D-AVE scan, motion artefacts caused by substructure deformation secondary to lung movement and heart contractions are present as aforementioned.

Although modern RT planning accounts for lung cancer movement associated with breathing, cardiac motion is not routinely accounted for during these processes. In a study using the maximum intensity projection (MIP) from 4D-CT, planning risk volume (PRV) margins of 5.8 mm and 4.8 mm were recommended for compensation of whole heart motion in the lateral and cranio-caudal axes [47]. However, independent displacement of the individual cardiac substructures is likely to be under-estimated by whole heart margins. Virtually any plane through the heart contains several substructures exhibiting non-synchronised and non-isotropic deformation patterns. Due to reciprocal compensation of motion, and buffering of exterior displacement by the surrounding pericardial lining, total displacement of substructures is therefore likely to be larger than the total heart margin. Furthermore, substructure motion will not necessarily be oscillatory after interactions from concurrent respiratory motion are considered. With gold standard cardiac-gated CT in breath-hold, where lung motion was obviated, centroid shifts for select substructures were limited to 0.5–1.6 mm [48].

Beyond cardiac motion, other unresolved issues in cardiac substructure segmentation include the lack of standardised great vessel definitions, as these are not defined in the published cardiac atlases [23], [42], [49], [50], [51]. Current atlases also do not recommend subtraction of the cardiac chamber blood pool, which is likely to be confounding how the dose metrics of the cardiac chamber muscles are interpreted [37], [46]. It will be crucial to have readily reproducible substructure definitions, given the pervasively difficult but clinically crucial task of reducing interobserver variation [52]. Similarly, there is a lack of guidance on how substructure studies should handle collinearity in the analysis of dose-volume statistics of intersecting structures such as the conduction system and atria, or the distal coronary arteries and myocardium.

Two alternate auto-segmentation algorithms that use a neural network architecture have been published, subsequent to the development of the tool presented, trained using 3D-CT planning cases. The model published by Garrett Fernandes et al had DSC of 0.74–0.95 across the same set of substructures and the largest median absolute difference in mean doses in the range 0.1 Gy–1.0 Gy [35]. The model published by Van Velzen et al had a DSC of 0.76–0.88 and R^2^ values for dosimetric parameters were 0.77–1.00 [53]. These values mirror those in the original description of the presented tool and in this updated 4D-CT test cohort, with an overall DSC of 0.76–0.93 and mean dose differences and R^2^ values of −1.6–0.3 Gy and 0.96–1.00. Taken together, the deep learning-based tool utilised in this study is suitable for use in 4D-CT RT planning scans, even though it was trained on a 3D-CT dataset.

The main strength of this study is that it is the first to apply what is the most widely applied substructure tool at the time of writing, a tool which is based on the most established cardiac atlas, and is widely applicable owing to its ‘open source’ availability, to 4D-CT. We are the second group to demonstrate the feasibility of cardiac substructure auto-segmentation on 4D-CT scans, and our results corroborate the findings of those investigators in that 4D-CT is a suitable planning scan modality for cardiac substructure auto-contouring [44], [54]. In those studies the mean DSC for the same structures was 0.88 [44] and 0.79 [54] across substructures, compared with our 0.85. Furthermore, this 4D-CT cohort included a blend of contrast-enhanced and non-contrast scans of patients with varying degrees of cardiac calcification.

The main limitations of this study are the cohort size and restriction to lung cancer cases. The 4D-AVE was used rather than the MIP despite prior work demonstrating the whole heart on 4D-AVE requires larger PRV margins than on MIP [47]. However, 4D-AVE was chosen for its superior soft tissue definition suited to delineating the complex geometries of the cardiac substructures, and for its similarity to the 3D-CT scan, on which our deep learning tool was based. DSC values for IVC and PA were relatively low compared to the other cardiac structures, but the dosimetric impact of this was low in this study, as has been found previously [55]. Although PRV margins have been proposed for the coronary arteries [56], none have been published for the cardiac substructures examined in this study and so these were not added. Finally, the auto-contouring tool has several minor weaknesses, largely related to ambiguity in the atlas on which the tool is based, as listed in Supplementary Table 10.

The development of auto-contouring tools for the cardiac substructures is warranted, because although atlases can reduce inter-observer variability (with or without contrast equally), the time required is significant. Moreover, rapid deep learning analysis made possible by robust tools such as the one presented could accelerate ‘big data’ analyses [36] such as interrogation of real-world data in substructure dose constraint investigations. Refinement of the historical clinical radiation response of the heart is required since the Gagliardi model involved limited endpoints and did not include patients with lung cancer [57]. These efforts should be multidisciplinary, spanning all thoracic tumor types, with aligned clinical endpoints and adjustment for relevant comorbidities.

## 5 Funding statement

This work was performed within the Irish Clinical Academic Training (ICAT) Programme, supported by the Wellcome Trust and the Health Research Board (Grant No 203930/B/16/Z), the Health Service Executive National Doctors Training and Planning and the Health and Social Care, Research and Development Division, Northern Ireland.

## Declaration of Competing Interest

The authors declare that they have no known competing financial interests or personal relationships that could have appeared to influence the work reported in this paper.
