# Supplementary material for: Validation of an established deep learning auto-segmentation tool for cardiac substructures in 4D radiotherapy planning scans
Source: Phys Imaging Radiat Oncol. 2022 Jul 26;23:118–26. doi: 10.1016/j.phro.2022.07.003 (PMC9356270; doi:10.1016/j.phro.2022.07.003)
Supplement: Supplementary data 1 [file mmc1.docx]

**Supplementary Data**

*Modification of Autocontours*

On clinical evaluation (CMC, GH), at least one imperfect slice were identified for 44/240 structures (18.3%), generally with a small number of slices that could be modified (median 2 slices, (range 1–8). For the purposes of the evaluations against manual contours in this study, the minor amendments required to correct these imperfections were implemented prior to commencing evaluation. These modifications were minor, resulting in a median change in structure volume of 1.6cc (range 0.0–18.7), and a median displacement of centroid of 0.3mm (range 0.0–19.3). The median DSC and HD95 comparing the modified and unmodified automated substructures were 0.9986 (range 0.9189–0.9998) and 1.88mm (range 0.00–20.40).
