# Supplementary material for: Validation of an established deep learning auto-segmentation tool for cardiac substructures in 4D radiotherapy planning scans
Source: Phys Imaging Radiat Oncol. 2022 Jul 26;23:118–26. doi: 10.1016/j.phro.2022.07.003 (PMC9356270; doi:10.1016/j.phro.2022.07.003)
Supplement: Supplementary data 2 [file mmc2.docx]

**Supp. Figure 1**


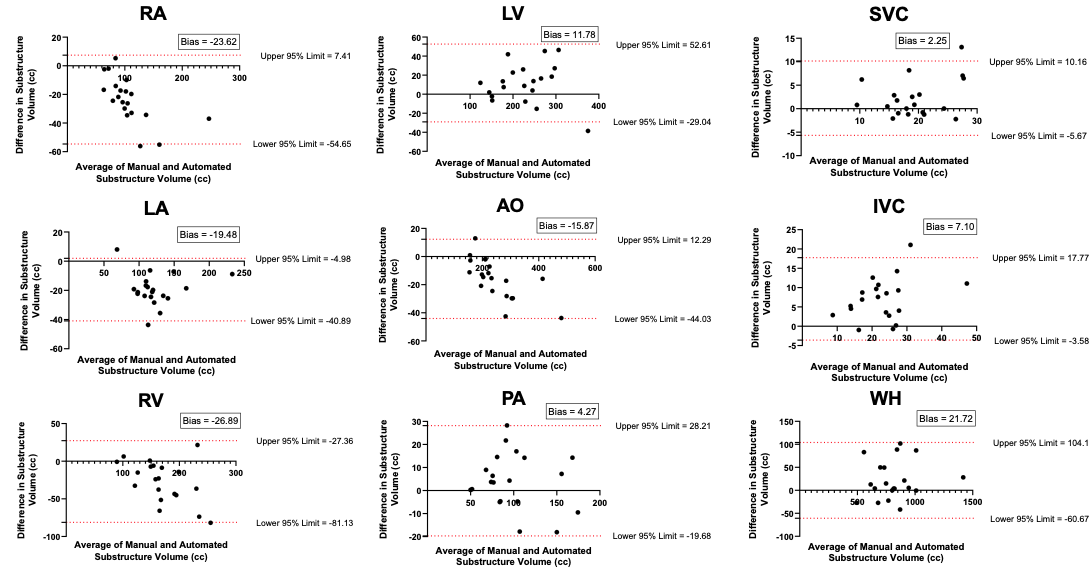


**Supp. Figure 1.** Bland-Altman plots for substructure volume calculated from automated and manual segmentation in 4D-AVE.

*(WH = whole heart; RA = right atrium; RV = right ventricle; LA = left atrium; LV = left ventricle; PA = pulmonary artery; SVC = superior vena cava; IVC = inferior vena cava; AO = aorta)*

**Supp. Figure 2**

**
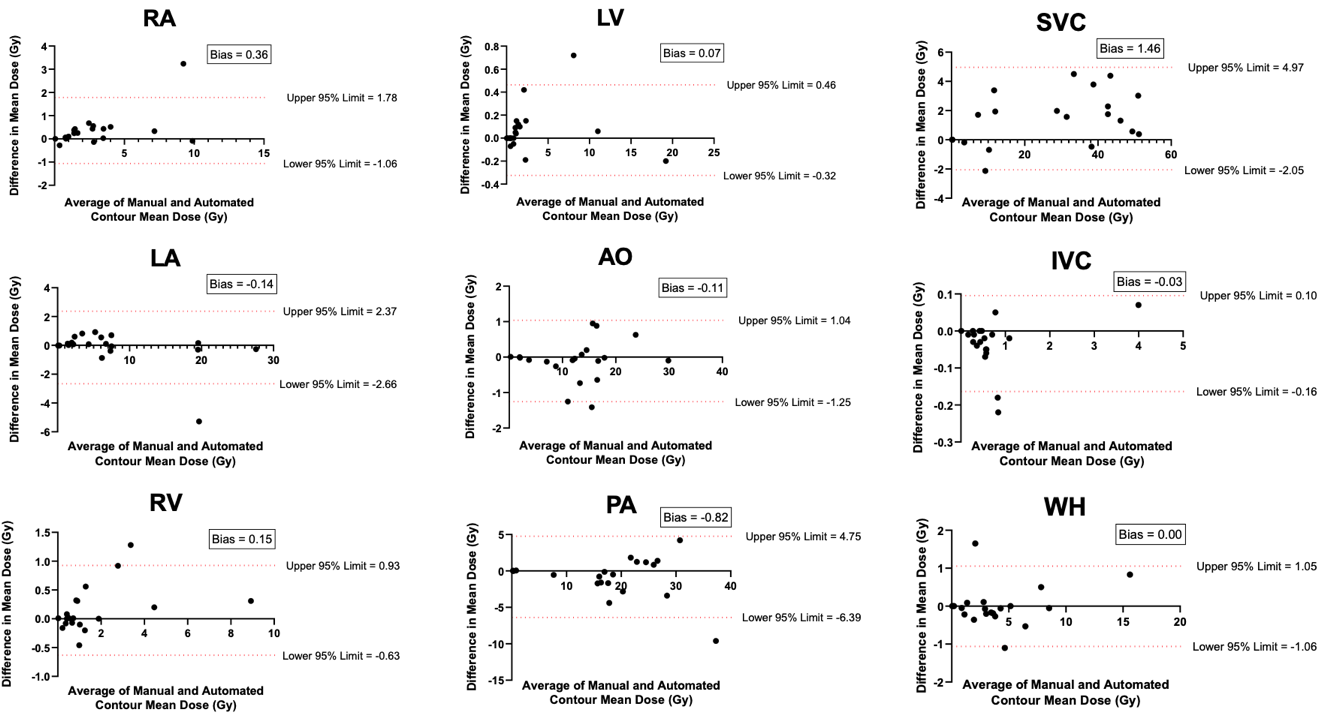
**

**Figure 2.** Bland-Altman plots for mean dose calculated from automated and manual segmentation in 4D-AVE scans.

*(WH = whole heart; RA = right atrium; RV = right ventricle; LA = left atrium; LV = left ventricle; PA = pulmonary artery; SVC = superior vena cava; IVC = inferior vena cava; AO = aorta)*

**Supp. Figure 3**

**
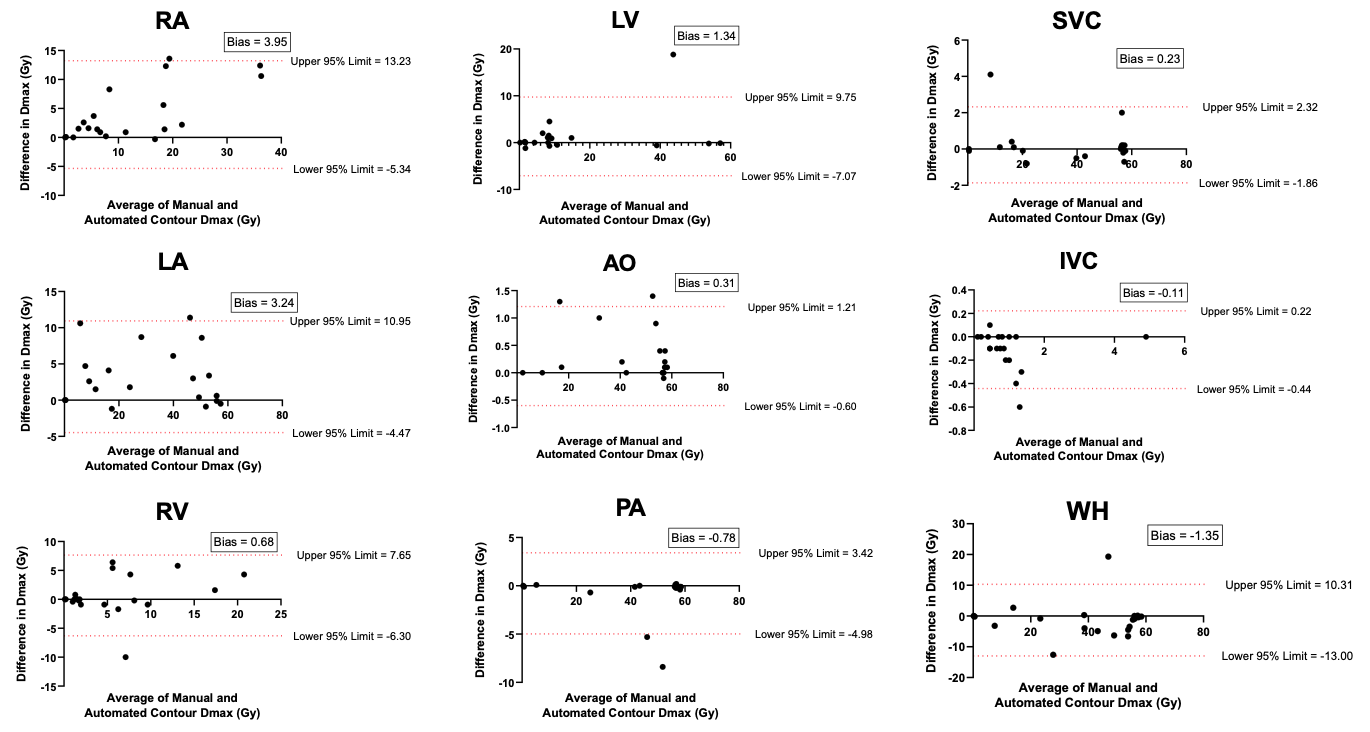
**

**Supp. Figure 3.** Bland-Altman plots for substructure Dmax (cc) calculated from automated and manual segmentation in 4D-AVE.

*(WH = whole heart; RA = right atrium; RV = right ventricle; LA = left atrium; LV = left ventricle; PA = pulmonary artery; SVC = superior vena cava; IVC = inferior vena cava; AO = aorta)*
