# Supplementary material for: Validation of an established deep learning auto-segmentation tool for cardiac substructures in 4D radiotherapy planning scans
Source: Phys Imaging Radiat Oncol. 2022 Jul 26;23:118–26. doi: 10.1016/j.phro.2022.07.003 (PMC9356270; doi:10.1016/j.phro.2022.07.003)
Supplement: Supplementary data 3 [file mmc3.docx]

**Supp. Table 1**

| **Scoring** | **Number of Slices Requiring Modification** | | | |
| --- | --- | --- | --- | --- |
|  | **Heart** | **Aorta** | **IVC** | **LA, RA, RV, SVC, PA** |
| **Good** | 0 | 0 | 0 | 0 |
| **Acceptable** | 1–8 | 1-10 | 1-3 | 1-5 |
| **Need of Adjustment** | 9–12 | 11-14 | 4-5 | 6-8 |
| **Poor** | >12 | >14 | >5 | >8 |

**Supp. Table 1.** Criteria for scoring each cardiac substructure contour in clinical scoring, showing the number of CT image slices that need to be modified for each level of quality (adopted from Table S1, Haq et al phiRO 2020)

*(IVC = inferior vena cava, LA = left atrium, LV = left ventricle, RA = right atrium; RV = right ventricle, SVC = superior vena cava; PA = pulmonary artery)*

**Supp. Table 2**

|  | **Number of Patients (%)** |
| --- | --- |
| **Female Gender** | 7 (35) |
| **Age (years)** | 70.5 |
| **Performance Status** | 1 |
| **Histology**  Squamous  Adenocarcinoma  Clinical  Small cell  NOS | 9 (45)  8 (40)  1 (5)  1 (5)  1 (5) |
| **T-Staging**  T0  T1  T2  T3  T4 | 1 (5)  5 (25)  8 (40)  2 (10)  4 (25) |
| **N-Staging**  N0  N1  N2  N3 | 6 (30)  4 (25)  8 (40)  2 (10) |
| **Dose Fractionation**  55Gy/20#  40Gy/15# | 19 (95)  1 (5) |
| **Intravenous Contrast**  Present  Absent | 14 (70)  6 (30) |
| **Planning Type**  VMAT | 20 (100) |
| **Algorithm**  AAA 13.6.23 | 20 (100) |
| **Coronary Calcification**  None  Mild  Moderate  Severe | 0  5 (25)  9 (45)  6 (30) |

**Supp. Table 2.** Clinical characteristics of the n=20 cohort.

**Supp. Table 3**

| **CASE** | **Contour** | **WH** | **PC** | **RA** | **LA** | **RV** | **LV** | **AO** | **PA** | **SVC** | **IVC** |
| --- | --- | --- | --- | --- | --- | --- | --- | --- | --- | --- | --- |
| **1** | **Manual** | 821.5 | 1311.9 | 91.5 | 129.2 | 147.6 | 281.1 | 502.1 | 179.2 | 15.5 | 7.3 |
|  | **Automated** | 923.4 | 1448.0 | 67.1 | 104.8 | 148.4 | 299.5 | 458.4 | 169.7 | 17.2 | 10.2 |
|  | **% Difference** | 12.4 | 10.4 | -26.7 | -18.9 | 0.5 | 6.6 | -8.7 | -5.3 | 11.4 | 39.6 |
| **2** | **Manual** | 1009.8 | 1195.5 | 187.8 | 146.4 | 221.2 | 283.1 | 211.4 | 115.9 | 24.3 | 20.5 |
|  | **Automated** | 1009.4 | 1224.5 | 132.8 | 122.7 | 242.8 | 329.6 | 209.6 | 98.1 | 24.4 | 41.6 |
|  | **% Difference** | 0.0 | 2.4 | -29.3 | -16.2 | 9.8 | 16.4 | -0.9 | -15.4 | 0.2 | 102.7 |
| **3** | **Manual** | 756.0 | 983.5 | 77.2 | 86.8 | 164.8 | 254.6 | 209.5 | 97.4 | 25.3 | 26.5 |
|  | **Automated** | 741.1 | 954.0 | 91.2 | 109.1 | 173.5 | 240.8 | 211.4 | 93.0 | 27.5 | 13.9 |
|  | **% Difference** | 2.0 | 3.1 | -15.4 | -20.4 | -5.0 | 5.7 | -0.9 | 4.6 | -8.1 | 90.4 |
| **4** | **Manual** | 801.7 | 1069.2 | 98.0 | 103.5 | 141.7 | 224.8 | 224.8 | 106.4 | 19.7 | 28.4 |
|  | **Automated** | 801.9 | 1016.1 | 107.9 | 121.2 | 193.1 | 232.5 | 240.1 | 78.1 | 18.9 | 19.9 |
|  | **% Difference** | 0.0 | 5.2 | -9.2 | -14.6 | -26.6 | -3.3 | -6.4 | 36.2 | 4.5 | 42.9 |
| **5** | **Manual** | 666.3 | 843.3 | 84.0 | 91.2 | 144.3 | 209.3 | 148.9 | 78.9 | 22.5 | 25.8 |
|  | **Automated** | 693.5 | 827.8 | 101.3 | 134.9 | 182.1 | 167.3 | 160.1 | 72.5 | 14.3 | 22.2 |
|  | **% Difference** | -3.9 | 1.9 | -17.1 | -32.3 | -20.7 | 25.1 | -7.0 | 8.8 | 57.0 | 16.2 |
| **6** | **Manual** | 1432.4 | 1937.0 | 228.2 | 228.4 | 211.8 | 355.6 | 405.8 | 175.3 | 30.9 | 52.7 |
|  | **Automated** | 1404.2 | 1771.0 | 265.1 | 237.2 | 248.2 | 394.1 | 421.6 | 161.1 | 24.5 | 41.7 |
|  | **% Difference** | 2.0 | 9.4 | -13.9 | -3.7 | -14.7 | -9.8 | -3.8 | 8.9 | 26.4 | 26.6 |
| **7** | **Manual** | 723.7 | 940.7 | 77.9 | 103.2 | 118.9 | 182.0 | 192.0 | 81.3 | 21.7 | 34.3 |
|  | **Automated** | 673.8 | 835.3 | 99.7 | 117.0 | 133.9 | 174.6 | 204.8 | 86.3 | 18.7 | 20.0 |
|  | **% Difference** | 7.4 | 12.6 | -21.8 | -11.8 | -11.2 | 4.3 | -6.2 | -5.8 | 16.1 | 71.2 |
| **8** | **Manual** | 888.2 | 1152.8 | 83.5 | 101.4 | 191.5 | 247.4 | 223.1 | 141.0 | 13.4 | 25.5 |
|  | **Automated** | 799.5 | 1042.8 | 109.0 | 118.1 | 205.6 | 243.4 | 247.6 | 159.1 | 7.2 | 17.9 |
|  | **% Difference** | 11.1 | 10.5 | -23.4 | -14.1 | -6.9 | 1.6 | -9.9 | -11.3 | 85.9 | 42.4 |
| **CASE** | **Contour** | **WH** | **PC** | **RA** | **LA** | **RV** | **LV** | **AO** | **PA** | **SVC** | **IVC** |
| **9** | **Manual** | 849.8 | 1081.6 | 91.8 | 107.4 | 172.0 | 230.7 | 185.0 | 102.0 | 20.2 | 16.5 |
|  | **Automated** | 891.5 | 1032.3 | 118.1 | 135.8 | 217.1 | 221.9 | 205.9 | 106.7 | 17.7 | 11.2 |
|  | **% Difference** | -4.7 | 4.8 | -22.2 | -20.9 | -20.8 | 4.0 | -10.1 | -4.5 | 14.3 | 46.5 |
| **10** | **Manual** | 917.6 | 1169.6 | 86.8 | 158.0 | 169.7 | 296.7 | 261.2 | 101.8 | 17.3 | 16.2 |
|  | **Automated** | 896.2 | 1129.5 | 121.4 | 176.6 | 213.2 | 251.4 | 303.7 | 80.0 | 14.4 | 11.7 |
|  | **% Difference** | 2.4 | 3.5 | -28.5 | -10.5 | -20.4 | 18.1 | -14.0 | 27.1 | 19.9 | 38.8 |
| **11** | **Manual** | 757.4 | 999.8 | 98.8 | 112.2 | 152.1 | 236.7 | 289.6 | 78.4 | 20.4 | 21.6 |
|  | **Automated** | 779.8 | 1036.3 | 154.9 | 147.8 | 174.8 | 210.7 | 319.3 | 74.9 | 21.1 | 12.8 |
|  | **% Difference** | -2.9 | -3.5 | -36.2 | -24.1 | -13.0 | 12.3 | -9.3 | 4.7 | -3.7 | 68.0 |
| **12** | **Manual** | 948.5 | 948.5 | 101.6 | 128.5 | 198.2 | 273.7 | 275.8 | 111.7 | 31.1 | 27.2 |
|  | **Automated** | 943.1 | 943.1 | 121.3 | 154.0 | 271.9 | 257.1 | 293.0 | 94.7 | 24.0 | 16.5 |
|  | **% Difference** | 0.6 | 0.6 | -16.3 | -16.5 | -27.1 | 6.5 | -5.9 | 18.0 | 29.3 | 65.1 |
| **13** | **Manual** | 477.9 | 665.1 | 55.1 | 87.6 | 89.2 | 130.1 | 215.6 | 52.0 | 14.6 | 15.7 |
|  | **Automated** | 504.9 | 641.9 | 71.8 | 108.8 | 90.0 | 118.2 | 227.4 | 51.4 | 16.7 | 16.7 |
|  | **% Difference** | -5.3 | 3.6 | -23.3 | -19.5 | -0.9 | 10.1 | -5.2 | 1.2 | -12.5 | -5.8 |
| **14** | **Manual** | 1052.6 | 1361.8 | 95.3 | 96.1 | 214.3 | 310.7 | 293.3 | 119.7 | 33.9 | 32.2 |
|  | **Automated** | 965.9 | 1270.5 | 128.2 | 119.9 | 296.1 | 283.5 | 322.9 | 105.4 | 20.8 | 22.9 |
|  | **% Difference** | 9.0 | 7.2 | -25.6 | -19.8 | -27.6 | 9.6 | -9.2 | 13.5 | 63.1 | 40.6 |
| **15** | **Manual** | 758.0 | 953.4 | 84.8 | 110.1 | 146.0 | 211.2 | 272.3 | 72.4 | 20.4 | 29.8 |
|  | **Automated** | 708.6 | 901.8 | 114.6 | 129.8 | 169.9 | 188.4 | 300.4 | 63.4 | 21.6 | 25.7 |
|  | **% Difference** | 7.0 | 5.7 | -26.0 | -15.2 | -14.1 | 12.1 | -9.3 | 14.1 | -5.7 | 15.7 |
| **16** | **Manual** | 653.6 | 823.4 | 86.7 | 83.1 | 132.2 | 183.1 | 195.8 | 88.1 | 18.0 | 20.6 |
|  | **Automated** | 649.6 | 826.5 | 81.3 | 102.3 | 198.0 | 169.6 | 210.3 | 73.5 | 17.9 | 13.7 |
|  | **% Difference** | 0.6 | -0.4 | 6.6 | -18.8 | -33.2 | 8.0 | -6.9 | 19.8 | 0.2 | 50.4 |
| **17** | **Manual** | 816.4 | 960.8 | 120.0 | 145.8 | 145.8 | 149.9 | 156.5 | 159.2 | 16.1 | 25.7 |
|  | **Automated** | 812.9 | 957.8 | 154.3 | 153.0 | 153.0 | 152.1 | 155.6 | 152.0 | 17.0 | 26.4 |
|  | **% Difference** | 0.4 | 0.3 | -22.2 | -4.7 | -4.7 | -1.5 | 0.6 | 4.8 | -5.6 | -2.7 |
| **CASE** | **Contour** | **WH** | **PC** | **RA** | **LA** | **RV** | **LV** | **AO** | **PA** | **SVC** | **IVC** |
| **18** | **Manual** | 823.5 | 1112.3 | 93.1 | 112.6 | 151.2 | 247.6 | 221.9 | 82.3 | 17.7 | 26.0 |
|  | **Automated** | 819.9 | 1019.3 | 110.9 | 119.0 | 157.2 | 263.0 | 229.0 | 86.9 | 18.9 | 16.4 |
|  | **% Difference** | 0.4 | 9.1 | -16.1 | -5.3 | -3.8 | -5.8 | -3.1 | -5.3 | -6.3 | 59.0 |
| **19** | **Manual** | 621.9 | 799.2 | 63.1 | 108.3 | 105.2 | 145.8 | 156.2 | 75.7 | 15.0 | 27.0 |
|  | **Automated** | 609.2 | 783.1 | 65.5 | 129.4 | 137.7 | 143.9 | 158.9 | 72.0 | 14.5 | 26.8 |
|  | **% Difference** | 2.1 | 2.1 | -3.7 | -16.3 | -23.6 | 1.3 | -1.7 | 5.2 | 3.4 | 0.8 |
| **20** | **Manual** | 598.3 | 687.9 | 70.7 | 72.6 | 104.7 | 148.3 | 181.5 | 50.1 | 9.9 | 26.3 |
|  | **Automated** | 515.3 | 611.7 | 72.7 | 64.6 | 98.4 | 154.9 | 168.5 | 49.8 | 9.1 | 23.5 |
|  | **% Difference** | 16.1 | 12.5 | -2.8 | 12.4 | 6.4 | -4.2 | 7.7 | 0.5 | 8.8 | 11.6 |
| **Median Volumes (cc)** | **Manual** | 802.1 | 1005.4 | 118.4 | 132.7 | 184.1 | 221.6 | 254.7 | 97.8 | 18.2 | 19.4 |
|  | **Automated** | 823.8 | 1058.1 | 94.8 | 113.2 | 157.2 | 233.4 | 238.8 | 102.1 | 20.5 | 26.5 |
|  | **Per Substructure**  **Median % Difference**  (range) | 1.32  (-5.3–16.1) | 4.19  (-3.5–12.6) | -22.01  (-36.2–6.6) | -16.23  (-32.3–12.4) | -13.56  (-33.2–9.8) | 6.08  (-9.8–25.1) | -6.31  (-14.0–7.7) | 4.72  (-15.4–36.2) | 6.63  (-12.5–85.9) | 41.47  (-5.8–102.7) |
|  | **p value** | 0.7180 | 0.5831 | 0.0195 | 0.0051 | 0.0634 | 0.6017 | 0.4135 | 0.4612 | 0.3443 | 0.0057 |

**Supp. Table 3.** Cardiac substructure volumes (cc) for manual and automated segmentation, with percentage differences relative to manual.

*(WH = whole heart; PC = pericardium; RA = right atrium; RV = right ventricle; LA = left atrium; LV = left ventricle; PA = pulmonary artery; SVC = superior vena cava; IVC = inferior vena cava; AO = aorta*

**Supp. Table 4**

| **Structure** | **Volume (cc)** | | |
| --- | --- | --- | --- |
|  | **r** | **95% CI** | **p value** |
| **WH** | 0.97 | 0.93–0.99 | <0.0001 |
| **PC** | 0.99 | 0.96–0.99 | <0.0001 |
| **RA** | 0.89 | 0.74–0.96 | <0.0001 |
| **LA** | 0.88 | 0.71–0.95 | <0.0001 |
| **RV** | 0.85 | 0.63–0.94 | <0.0001 |
| **LV** | 0.97 | 0.93–0.99 | <0.0001 |
| **AO** | 0.99 | 0.96–0.99 | <0.0001 |
| **PA** | 0.91 | 0.77–0.96 | <0.0001 |
| **SVC** | 0.78 | 0.51–0.91 | <0.0001 |
| **IVC** | 0.68 | 0.33–0.87 | 0.001 |

**Supp. Table 4.** Spearman’s correlation coefficients for the comparison of automated and manual segmentation volumes for each cardiac substructure.

*(PC = pericardium; WH = whole heart; RA = right atrium; RV = right ventricle; LA = left atrium; LV = left ventricle; PA = pulmonary artery; SVC = superior vena cava; IVC = inferior vena cava; AO = aorta)*

**Supp. Table 5**

| **CASE ID** | **WH** | **PC** | **RA** | **LA** | **RV** | **LV** | **AO** | **PA** | **SVC** | **IVC** |
| --- | --- | --- | --- | --- | --- | --- | --- | --- | --- | --- |
| **1** | 0.9256 | 0.9101 | 0.7276 | 0.7915 | 0.8124 | 0.9106 | 0.9334 | 0.9271 | 0.7802 | 0.4864 |
| **2** | 0.9515 | 0.9525 | 0.8078 | 0.8018 | 0.8224 | 0.8739 | 0.9304 | 0.7624 | 0.8600 | 0.6226 |
| **3** | 0.9489 | 0.9400 | 0.8126 | 0.8353 | 0.8660 | 0.8863 | 0.9209 | 0.8620 | 0.8206 | 0.6551 |
| **4** | 0.9384 | 0.9387 | 0.8204 | 0.8710 | 0.8069 | 0.9110 | 0.9294 | 0.8133 | 0.8081 | 0.7204 |
| **5** | 0.9500 | 0.9400 | 0.8015 | 0.7746 | 0.8622 | 0.8469 | 0.9263 | 0.8767 | 0.6911 | 0.7670 |
| **6** | 0.9367 | 0.9242 | 0.8803 | 0.9015 | 0.8180 | 0.9042 | 0.9455 | 0.8658 | 0.8011 | 0.8688 |
| **7** | 0.9260 | 0.9142 | 0.8173 | 0.8913 | 0.8464 | 0.8824 | 0.9486 | 0.8593 | 0.7798 | 0.7199 |
| **8** | 0.9314 | 0.9118 | 0.7733 | 0.8085 | 0.8743 | 0.8876 | 0.9117 | 0.8694 | 0.5687 | 0.7598 |
| **9** | 0.9210 | 0.9384 | 0.7940 | 0.8480 | 0.8220 | 0.9083 | 0.8946 | 0.8833 | 0.7616 | 0.7647 |
| **10** | 0.9553 | 0.9529 | 0.7423 | 0.8680 | 0.8166 | 0.8869 | 0.9151 | 0.8077 | 0.7372 | 0.6913 |
| **11** | 0.9504 | 0.9386 | 0.7735 | 0.8238 | 0.7855 | 0.8739 | 0.9400 | 0.7939 | 0.8716 | 0.6313 |
| **12** | 0.9285 | 0.9285 | 0.8187 | 0.8428 | 0.8018 | 0.9050 | 0.9471 | 0.8246 | 0.8415 | 0.7285 |
| **13** | 0.9177 | 0.9492 | 0.8181 | 0.8221 | 0.8214 | 0.8788 | 0.9333 | 0.8726 | 0.8335 | 0.8712 |
| **14** | 0.9154 | 0.9122 | 0.7615 | 0.8149 | 0.7895 | 0.8790 | 0.9330 | 0.8476 | 0.6992 | 0.7943 |
| **15** | 0.9291 | 0.9282 | 0.8161 | 0.8881 | 0.8356 | 0.9029 | 0.9405 | 0.8647 | 0.7770 | 0.8868 |
| **16** | 0.9380 | 0.9366 | 0.7147 | 0.8402 | 0.7709 | 0.9144 | 0.9449 | 0.7990 | 0.7709 | 0.7400 |
| **17** | 0.9451 | 0.9434 | 0.8586 | 0.9167 | 0.7980 | 0.8981 | 0.9296 | 0.8070 | 0.8431 | 0.9012 |
| **18** | 0.9140 | 0.8999 | 0.8767 | 0.8925 | 0.8552 | 0.9067 | 0.9498 | 0.8712 | 0.8849 | 0.8933 |
| **19** | 0.9411 | 0.9368 | 0.8687 | 0.8873 | 0.8372 | 0.9235 | 0.9370 | 0.9202 | 0.7949 | 0.9146 |
| **20** | 0.9102 | 0.9116 | 0.8607 | 0.8502 | 0.8531 | 0.9094 | 0.9114 | 0.7493 | 0.7550 | 0.8531 |
| **Per Substructure Median DSC**  (range) | 0.93  (0.91–0.96) | 0.94  (0.90–0.95) | 0.81  (0.71–0.88) | 0.85  (0.77–0.88) | 0.82  (0.77–0.87) | 0.90 (0.85–0.92) | 0.93  (0.89–0.95) | 0.86  (0.75–0.93) | 0.79  (0.57–0.88) | 0.76  (0.49–0.91) |

**Supp. Table 5.** Cardiac substructure Dice Similarity Coefficients comparing manual and automated segmentation, with averages per substructure (bottom row).

*(DSC = Dice similarity coefficient; WH = whole heart; PC = pericardium; RA = right atrium; RV = right ventricle; LA = left atrium; LV = left ventricle; PA = pulmonary artery; SVC = superior vena cava; IVC = inferior vena cava; AO = aorta)*

**Supp. Table 6**

| **Case ID** | **WH** | **PC** | **RA** | **LA** | **RV** | **LV** | **AO** | **PA** | **SVC** | **IVC** |
| --- | --- | --- | --- | --- | --- | --- | --- | --- | --- | --- |
| **1** | 3.0 | 4.6 | 9.3 | 6.4 | 6.6 | 3.5 | 1.6 | 0.8 | 2.7 | 8.1 |
| **2** | 0.6 | 1.2 | 5.8 | 8.0 | 5.7 | 4.9 | 0.8 | 17.7 | 3.8 | 11.4 |
| **3** | 1.9 | 3.4 | 3.7 | 4.2 | 1.4 | 3.4 | 4.9 | 8.7 | 6.3 | 4.7 |
| **4** | 1.6 | 2.0 | 3.8 | 1.9 | 5.0 | 2.8 | 1.7 | 4.8 | 6.5 | 2.9 |
| **5** | 0.9 | 2.3 | 4.3 | 7.2 | 2.1 | 6.1 | 3.9 | 2.7 | 1.9 | 3.5 |
| **6** | 2.7 | 3.3 | 2.8 | 2.6 | 4.1 | 1.4 | 0.5 | 7.9 | 6.5 | 2.0 |
| **7** | 1.5 | 2.6 | 3.9 | 2.1 | 2.2 | 3.3 | 1.0 | 6.2 | 7.3 | 4.7 |
| **8** | 1.7 | 4.1 | 3.5 | 4.7 | 2.9 | 3.7 | 4.1 | 2.9 | 3.9 | 5.6 |
| **9** | 5.7 | 3.5 | 1.9 | 0.8 | 2.8 | 2.5 | 2.6 | 4.7 | 1.8 | 4.5 |
| **10** | 2.0 | 2.2 | 3.3 | 2.6 | 6.6 | 4.0 | 2.8 | 5.3 | 4.9 | 4.4 |
| **11** | 1.1 | 3.8 | 3.6 | 5.7 | 9.3 | 3.7 | 0.8 | 8.9 | 1.3 | 5.5 |
| **12** | 2.9 | 2.9 | 4.2 | 1.3 | 5.2 | 3.1 | 1.3 | 8.5 | 1.9 | 4.9 |
| **13** | 4.2 | 2.3 | 4.0 | 3.9 | 5.8 | 4.8 | 1.9 | 1.6 | 2.1 | 1.0 |
| **14** | 3.0 | 5.3 | 3.8 | 3.0 | 7.8 | 3.6 | 1.0 | 7.4 | 5.1 | 2.8 |
| **15** | 1.9 | 2.3 | 2.3 | 1.4 | 5.1 | 2.4 | 1.7 | 3.3 | 8.0 | 2.9 |
| **16** | 2.0 | 4.3 | 7.8 | 2.4 | 7.1 | 1.9 | 1.6 | 9.1 | 11.4 | 7.2 |
| **17** | 1.3 | 1.7 | 2.9 | 0.5 | 0.5 | 6.9 | 5.0 | 2.8 | 3.9 | 1.6 |
| **18** | 1.6 | 2.7 | 1.0 | 2.2 | 2.6 | 2.8 | 1.1 | 6.6 | 2.0 | 0.6 |
| **19** | 1.2 | 2.9 | 2.2 | 1.5 | 1.8 | 1.3 | 3.2 | 2.8 | 9.3 | 1.1 |
| **20** | 4.7 | 3.4 | 1.8 | 2.2 | 3.6 | 1.7 | 5.3 | 6.0 | 5.8 | 1.9 |
| **Per Substructure Median CS in millimetres**  (range) | 1.9  (0.6–5.7) | 2.9  (1.2–5.3) | 3.7  (1.0–7.8) | 2.5  (0.5–7.2) | 4.6  (0.5–9.3) | 3.4  (1.3–6.9) | 1.7  (0.5–5.3) | 5.7  (1.6–9.1) | 4.4  (1.3–11.4) | 4.0  (0.6–7.2) |

**Supp. Table 6.** Cardiac substructure centroid shifts comparing manual and automated segmentation, with averages per substructure (bottom row).

*(CS = centroid shift; WH = whole heart; PC = pericardium; RA = right atrium; RV = right ventricle; LA = left atrium; LV = left ventricle; PA = pulmonary artery; SVC = superior vena cava; IVC = inferior vena cava; AO = aorta)*

**Supp. Table 7.**

| **Substructure** | **Median Shift in Centroid Across All Cases (mm)** | | |
| --- | --- | --- | --- |
|  | **X** | **Y** | **Z** |
| **WH** | -0.50 | -1.25 | 0.66 |
| **PC** | 0.24 | -1.90 | 1.31 |
| **RA** | -0.34 | -1.59 | 0.61 |
| **LA** | -1.95 | -0.34 | -0.59 |
| **RV** | -1.59 | 0.70 | 2.24 |
| **LV** | -2.48 | 1.06 | 0.28 |
| **AO** | -0.33 | 0.40 | -0.68 |
| **PA** | -0.80 | 0.18 | 0.28 |
| **SVC** | -0.18 | 0.01 | 1.70 |
| **IVC** | 0.11 | -0.78 | -2.24 |
| **Overall Median Shift** | **-0.42** | **-0.17** | **0.44** |

**Supp. Table 7.** Median cardiac substructure centroid shifts in three dimensions comparing manual and automated segmentation, with averages per direction (bottom row).

*(WH = whole heart; PC = pericardium; RA = right atrium; RV = right ventricle; LA = left atrium; LV = left ventricle; PA = pulmonary artery; SVC = superior vena cava; IVC = inferior vena cava; AO = aorta)*

**Supp. Table 8**

| **Structure** | **95^th^ Percentile Hausdorff Distance (mm)** |
| --- | --- |
| **WH** | 6.8 (3.1–14.4) |
| **PC** | 6.5 (4.7–11.3) |
| **Atria** | 9.1 (5.7–15.6) |
| **Ventricles** | 7.5 (5.3–13.9) |
| **RA** | 8.4 (4.6–14.8) |
| **LA** | 8.0 (4.5–12.2) |
| **RV** | 8.9 (5.1–14.0) |
| **LV** | 6.5 (4.3–10.9) |
| **AO** | 2.4 (1.8–8.9) |
| **PA** | 6.1 (3.5–14.1) |
| **SVC** | 7.4 (3.2–12.6) |
| **IVC** | 6.3 (2.5–13.2) |
| **Median Absolute Value Across All Substructures in 4D-CT Dataset**  (range) | 7.1  (2.4–9.1) |
| **Median Across All Substructures in 3D-CT Dataset** (1)  (range) | 5.9  (3.1–7.3) |

**Supp. Table 8.** Geometric comparison of the automated and manual segmentation in 4D-AVE scans by 95^th^ percentile of the Hausdorff distance, and comparison of overall performance with published data for 3D-CT scans. All averages are median values.

*(3D = three-dimensional; 4D = four-dimensional; WH = whole heart; PC = pericardium; RA = right atrium; RV = right ventricle; LA = left atrium; LV = left ventricle; PA = pulmonary artery; SVC = superior vena cava; IVC = inferior vena cava; AO = aorta)*

**Supp. Table 9**

| **CASE** | **Contour** | **WH** | **PC** | **RA** | **LA** | **RV** | **LV** | **AO** | **PA** | **SVC** | **IVC** |
| --- | --- | --- | --- | --- | --- | --- | --- | --- | --- | --- | --- |
| **1** | **Manual** | 0.19 | 0.98 | 0.22 | 0.25 | 0.14 | 0.13 | 2.03 | 0.82 | 3.38 | 0.16 |
|  | **Automated** | 0.19 | 1.01 | 0.50 | 0.26 | 0.30 | 0.13 | 2.05 | 0.77 | 3.56 | 0.17 |
|  | **Difference** | 0.00 | 0.03 | 0.28 | 0.01 | 0.16 | 0.00 | 0.02 | -0.05 | 0.18 | 0.01 |
| **2** | **Manual** | 6.18 | 12.54 | 2.74 | 17.01 | 0.76 | 2.32 | 29.80 | 32.50 | 38.00 | 0.73 |
|  | **Automated** | 6.71 | 12.78 | 2.88 | 22.29 | 1.22 | 1.90 | 29.90 | 42.10 | 38.47 | 0.95 |
|  | **Difference** | 0.53 | 0.24 | 0.14 | 5.28 | 0.46 | -0.42 | 0.10 | 9.60 | 0.47 | 0.22 |
| **3** | **Manual** | 2.92 | 10.94 | 1.78 | 7.83 | 0.72 | 1.10 | 24.06 | 26.67 | 40.65 | 0.80 |
|  | **Automated** | 3.12 | 10.14 | 1.53 | 7.13 | 0.71 | 1.05 | 23.43 | 30.07 | 36.86 | 0.75 |
|  | **Difference** | 0.20 | -0.80 | -0.25 | -0.70 | -0.01 | -0.05 | -0.63 | 3.40 | -3.79 | -0.05 |
| **4** | **Manual** | 3.52 | 9.08 | 3.05 | 6.33 | 1.57 | 1.49 | 16.64 | 23.47 | 45.57 | 0.52 |
|  | **Automated** | 3.71 | 9.13 | 2.49 | 5.78 | 1.01 | 1.37 | 16.75 | 22.23 | 41.18 | 0.54 |
|  | **Difference** | 0.19 | 0.05 | -0.56 | -0.55 | -0.56 | -0.12 | 0.11 | -1.24 | -4.39 | 0.02 |
| **5** | **Manual** | 1.39 | 5.05 | 1.59 | 2.23 | 0.49 | 0.45 | 10.38 | 15.61 | 32.24 | 0.47 |
|  | **Automated** | 1.30 | 5.50 | 1.21 | 2.16 | 0.47 | 0.52 | 11.63 | 16.38 | 30.67 | 0.47 |
|  | **Difference** | -0.09 | 0.45 | -0.38 | -0.07 | -0.02 | 0.07 | 1.25 | 0.77 | -1.57 | 0.00 |
| **6** | **Manual** | 2.85 | 5.83 | 2.80 | 4.37 | 0.97 | 1.27 | 8.63 | 14.83 | 29.78 | 0.42 |
|  | **Automated** | 2.74 | 5.85 | 2.12 | 4.29 | 1.07 | 1.12 | 8.89 | 16.54 | 27.80 | 0.45 |
|  | **Difference** | -0.11 | 0.02 | -0.68 | -0.08 | 0.10 | -0.15 | 0.26 | 1.71 | -1.98 | 0.03 |
| **7** | **Manual** | 2.88 | 5.67 | 1.52 | 5.68 | 0.63 | 1.11 | 11.86 | 15.49 | 8.17 | 0.42 |
|  | **Automated** | 2.95 | 5.69 | 1.28 | 4.76 | 0.70 | 1.02 | 11.95 | 17.09 | 10.30 | 0.42 |
|  | **Difference** | 0.07 | 0.02 | -0.24 | -0.92 | 0.07 | -0.09 | 0.09 | 1.60 | 2.13 | 0.00 |
| **8** | **Manual** | 4.25 | 4.51 | 7.32 | 5.73 | 1.89 | 2.19 | 3.69 | 7.39 | 13.36 | 0.51 |
|  | **Automated** | 4.31 | 4.28 | 6.99 | 6.60 | 1.89 | 2.38 | 3.77 | 7.94 | 9.97 | 0.58 |
|  | **Difference** | 0.06 | -0.23 | -0.33 | 0.87 | 0.00 | 0.19 | 0.08 | 0.55 | -3.39 | 0.07 |
| **9** | **Manual** | 2.89 | 6.82 | 1.05 | 2.07 | 0.42 | 0.55 | 16.85 | 18.25 | 43.85 | 0.34 |
|  | **Automated** | 1.24 | 6.61 | 0.95 | 1.88 | 0.42 | 0.55 | 15.97 | 18.75 | 41.56 | 0.38 |
|  | **Difference** | -1.65 | -0.21 | -0.10 | -0.19 | 0.00 | 0.00 | -0.88 | 0.50 | -2.29 | 0.04 |
| **CASE** | **Contour** | **WH** | **PC** | **RA** | **LA** | **RV** | **LV** | **AO** | **PA** | **SVC** | **IVC** |
| **10** | **Manual** | 3.36 | 8.74 | 1.68 | 6.77 | 1.01 | 1.16 | 16.21 | 25.12 | 46.79 | 0.55 |
|  | **Automated** | 3.53 | 8.58 | 1.25 | 6.67 | 0.69 | 1.12 | 16.85 | 23.93 | 45.48 | 0.60 |
|  | **Difference** | 0.17 | -0.16 | -0.43 | -0.10 | -0.32 | -0.04 | 0.64 | -1.19 | -1.31 | 0.05 |
| **11** | **Manual** | 0.85 | 6.71 | 0.81 | 1.53 | 0.46 | 0.44 | 12.29 | 22.66 | 43.58 | 0.26 |
|  | **Automated** | 0.90 | 6.18 | 0.75 | 1.51 | 0.38 | 0.44 | 12.33 | 20.83 | 41.83 | 0.29 |
|  | **Difference** | 0.05 | -0.53 | -0.06 | -0.02 | -0.08 | 0.00 | 0.04 | -1.83 | -1.75 | 0.03 |
| **12** | **Manual** | 0.05 | 0.12 | 0.05 | 0.07 | 0.03 | 0.03 | 0.29 | 0.22 | 0.25 | 0.01 |
|  | **Automated** | 0.05 | 0.12 | 0.05 | 0.08 | 0.02 | 0.03 | 0.28 | 0.20 | 0.25 | 0.01 |
|  | **Difference** | 0.00 | 0.00 | 0.00 | 0.01 | -0.01 | 0.00 | -0.01 | -0.02 | 0.00 | 0.00 |
| **13** | **Manual** | 5.15 | 4.09 | 3.50 | 3.84 | 4.56 | 11.04 | 1.96 | 0.30 | 0.20 | 1.08 |
|  | **Automated** | 5.15 | 4.01 | 3.47 | 3.02 | 4.36 | 10.98 | 1.96 | 0.31 | 0.19 | 1.10 |
|  | **Difference** | 0.00 | -0.08 | -0.03 | -0.82 | -0.20 | -0.06 | 0.00 | 0.01 | -0.01 | 0.02 |
| **14** | **Manual** | 8.08 | 8.53 | 3.72 | 19.63 | 4.01 | 8.45 | 6.99 | 16.88 | 8.10 | 0.54 |
|  | **Automated** | 7.58 | 7.76 | 3.29 | 19.47 | 2.73 | 7.73 | 7.12 | 17.01 | 6.39 | 0.60 |
|  | **Difference** | -0.50 | -0.77 | -0.43 | -0.16 | -1.28 | -0.72 | 0.13 | 0.13 | -1.71 | 0.06 |
| **15** | **Manual** | 3.67 | 9.72 | 2.94 | 7.46 | 1.06 | 0.82 | 13.65 | 26.36 | 52.48 | 0.54 |
|  | **Automated** | 3.94 | 9.51 | 2.51 | 7.52 | 0.75 | 0.87 | 13.58 | 25.52 | 49.46 | 0.59 |
|  | **Difference** | 0.27 | -0.21 | -0.43 | 0.06 | -0.31 | 0.05 | -0.07 | -0.84 | -3.02 | 0.05 |
| **16** | **Manual** | 8.50 | 13.71 | 10.85 | 19.35 | 3.24 | 2.41 | 17.86 | 32.83 | 51.37 | 0.74 |
|  | **Automated** | 8.55 | 12.60 | 7.61 | 19.65 | 2.32 | 2.26 | 17.88 | 28.63 | 50.98 | 0.92 |
|  | **Difference** | 0.05 | -1.11 | -3.24 | 0.30 | -0.92 | -0.15 | 0.02 | -4.20 | -0.39 | 0.18 |
| **17** | **Manual** | 1.01 | 6.21 | 0.89 | 1.47 | 0.33 | 0.41 | 14.79 | 15.60 | 35.68 | 0.27 |
|  | **Automated** | 1.23 | 6.51 | 0.86 | 1.35 | 0.41 | 0.41 | 16.20 | 20.00 | 31.18 | 0.27 |
|  | **Difference** | 0.22 | 0.30 | -0.03 | -0.12 | 0.08 | 0.00 | 1.41 | 4.40 | -4.50 | 0.00 |
| **18** | **Manual** | 4.10 | 8.85 | 4.27 | 7.14 | 1.15 | 1.66 | 14.62 | 18.88 | 49.68 | 0.70 |
|  | **Automated** | 5.20 | 9.06 | 3.75 | 7.53 | 1.35 | 1.56 | 14.42 | 21.70 | 49.11 | 0.71 |
|  | **Difference** | 1.10 | 0.21 | -0.52 | 0.39 | 0.20 | -0.10 | -0.20 | 2.82 | -0.57 | 0.01 |
| **19** | **Manual** | 1.77 | 7.57 | 0.82 | 2.67 | 0.63 | 0.86 | 12.92 | 27.32 | 12.92 | 0.29 |
|  | **Automated** | 2.13 | 7.63 | 0.81 | 2.07 | 0.64 | 0.86 | 13.65 | 25.93 | 10.98 | 0.30 |
|  | **Difference** | 0.36 | 0.06 | -0.01 | -0.60 | 0.01 | 0.00 | 0.73 | -1.39 | -1.94 | 0.01 |
| **CASE** | **Contour** | **WH** | **PC** | **RA** | **LA** | **RV** | **LV** | **AO** | **PA** | **SVC** | **IVC** |
| **20** | **Manual** | 16.02 | 15.01 | 9.83 | 27.44 | 9.08 | 19.10 | 16.16 | 16.77 | 9.91 | 4.03 |
|  | **Automated** | 15.19 | 14.12 | 9.92 | 27.70 | 8.77 | 19.30 | 15.21 | 18.44 | 10.60 | 3.96 |
|  | **Difference** | -0.83 | -0.89 | 0.09 | 0.26 | -0.31 | 0.20 | -0.95 | 1.67 | 0.69 | -0.07 |
| **Median Mean Dose (cc)** | **Manual** | 3.98 | 7.53 | 3.07 | 7.44 | 1.66 | 2.85 | 12.58 | 17.90 | 28.30 | 0.67 |
|  | **Automated** | 3.99 | 7.35 | 2.71 | 7.59 | 1.51 | 2.78 | 12.69 | 18.72 | 26.84 | 0.70 |
|  | **Per Substructure Median Difference in Mean Dose**  (range) | 0.06 (-0.11–1.10) | -0.04  (-1.11–0.45) | -0.25  (-3.24–0.28) | -0.08  (-0.92–5.28) | -0.01  (-1.28–0.46) | -0.02  (-0.72–0.20) | 0.06  (-0.95–1.41) | 0.32  (-4.20–9.60) | -1.64  (-4.50–2.13) | 0.02  (-0.07–0.22) |
|  | **p value** | 0.8883 | 0.9526 | 0.6158 | 0.9949 | 0.8883 | 0.8989 | 0.9307 | 0.7180 | 0.7234 | 0.6155 |

**Supp. Table 9.** Cardiac substructure mean dose (Gy) for manual and automated segmentation, with differences relative to manual.

*(PC = pericardium; WH = whole heart; RA = right atrium; RV = right ventricle; LA = left atrium; LV = left ventricle; PA = pulmonary artery; SVC = superior vena cava; IVC = inferior vena cava; AO = aorta)*

**Supp. Table 10**

| **CASE** | **Contour** | **WH** | **PC** | **RA** | **LA** | **RV** | **LV** | **AO** | **PA** | **SVC** | **IVC** |
| --- | --- | --- | --- | --- | --- | --- | --- | --- | --- | --- | --- |
| **1** | **Manual** | 0.40 | 12.20 | 0.40 | 0.40 | 0.20 | 0.20 | 9.80 | 5.30 | 11.80 | 0.20 |
|  | **Automated** | 0.60 | 11.80 | 0.30 | 0.40 | 0.20 | 0.20 | 9.80 | 5.20 | 11.70 | 0.20 |
|  | **Difference** | 0.20 | -0.40 | -0.10 | 0.00 | 0.00 | 0.00 | 0.00 | -0.10 | -0.10 | 0.00 |
| **2** | **Manual** | 57.20 | 58.46 | 19.20 | 57.10 | 2.10 | 53.10 | 58.30 | 57.30 | 56.90 | 1.00 |
|  | **Automated** | 57.60 | 58.50 | 17.80 | 57.60 | 12.10 | 34.30 | 58.20 | 57.40 | 57.60 | 1.60 |
|  | **Difference** | 0.40 | 0.04 | -1.40 | 0.50 | 10.00 | -18.80 | -0.10 | 0.10 | 0.70 | 0.60 |
| **3** | **Manual** | 54.90 | 57.20 | 7.30 | 54.70 | 1.60 | 8.60 | 56.90 | 56.90 | 56.20 | 1.00 |
|  | **Automated** | 56.10 | 57.20 | 3.60 | 46.10 | 1.80 | 7.50 | 56.90 | 57.10 | 56.10 | 1.40 |
|  | **Difference** | 1.20 | 0.00 | -3.70 | -8.60 | 0.20 | -1.10 | 0.00 | 0.20 | -0.10 | 0.40 |
| **4** | **Manual** | 40.80 | 58.50 | 21.10 | 24.90 | 9.80 | 9.60 | 57.50 | 58.40 | 57.50 | 0.80 |
|  | **Automated** | 45.70 | 58.50 | 15.50 | 23.10 | 5.50 | 8.70 | 57.40 | 58.50 | 57.30 | 1.00 |
|  | **Difference** | 4.90 | 0.00 | -5.60 | -1.80 | -4.30 | -0.90 | -0.10 | 0.10 | -0.20 | 0.20 |
| **5** | **Manual** | 15.30 | 43.40 | 7.80 | 10.40 | 1.48 | 1.10 | 42.40 | 43.30 | 42.70 | 0.80 |
|  | **Automated** | 12.60 | 43.40 | 7.60 | 7.80 | 1.35 | 2.30 | 42.40 | 43.30 | 43.10 | 0.80 |
|  | **Difference** | -2.70 | 0.00 | -0.20 | -2.60 | -0.13 | 1.20 | 0.00 | 0.00 | 0.40 | 0.00 |
| **6** | **Manual** | 36.70 | 41.60 | 24.90 | 32.60 | 4.20 | 7.60 | 40.80 | 41.50 | 39.50 | 0.70 |
|  | **Automated** | 40.70 | 41.70 | 12.60 | 23.90 | 5.10 | 5.60 | 40.60 | 41.60 | 40.00 | 0.70 |
|  | **Difference** | 4.00 | 0.10 | -12.30 | -8.70 | 0.90 | -2.00 | -0.20 | 0.10 | 0.50 | 0.00 |
| **7** | **Manual** | 51.60 | 56.80 | 6.80 | 51.80 | 1.50 | 10.80 | 54.30 | 56.50 | 10.40 | 0.60 |
|  | **Automated** | 56.10 | 56.90 | 5.40 | 40.40 | 2.41 | 6.30 | 53.40 | 56.70 | 6.30 | 0.70 |
|  | **Difference** | 4.50 | 0.10 | -1.40 | -11.40 | 0.91 | -4.50 | -0.90 | 0.20 | -4.10 | 0.10 |
| **8** | **Manual** | 22.90 | 25.80 | 22.80 | 16.80 | 8.00 | 8.20 | 17.20 | 24.80 | 21.20 | 0.80 |
|  | **Automated** | 23.70 | 24.90 | 20.60 | 18.00 | 8.20 | 8.20 | 15.90 | 25.50 | 22.00 | 0.90 |
|  | **Difference** | 0.80 | -0.90 | -2.20 | 1.20 | 0.20 | 0.00 | -1.30 | 0.70 | 0.80 | 0.10 |
| **CASE** | **Contour** | **WH** | **PC** | **RA** | **LA** | **RV** | **LV** | **AO** | **PA** | **SVC** | **IVC** |
| **9** | **Manual** | 56.60 | 57.50 | 4.90 | 12.20 | 1.20 | 1.70 | 57.30 | 56.90 | 56.80 | 0.40 |
|  | **Automated** | 37.30 | 57.50 | 2.30 | 10.70 | 1.20 | 1.70 | 57.20 | 56.70 | 57.00 | 0.50 |
|  | **Difference** | -19.30 | 0.00 | -2.60 | -1.50 | 0.00 | 0.00 | -0.10 | -0.20 | 0.20 | 0.10 |
| **10** | **Manual** | 58.30 | 58.50 | 12.50 | 54.80 | 8.80 | 10.50 | 57.40 | 58.00 | 57.40 | 0.70 |
|  | **Automated** | 58.50 | 58.50 | 4.20 | 51.40 | 2.40 | 11.00 | 57.20 | 58.40 | 55.40 | 0.80 |
|  | **Difference** | 0.20 | 0.00 | -8.30 | -3.40 | -6.40 | 0.50 | -0.20 | 0.40 | -2.00 | 0.10 |
| **11** | **Manual** | 5.90 | 58.80 | 3.40 | 11.10 | 1.70 | 1.60 | 57.60 | 58.40 | 56.80 | 0.40 |
|  | **Automated** | 9.10 | 58.80 | 1.90 | 0.50 | 0.90 | 1.50 | 57.20 | 58.50 | 56.60 | 0.40 |
|  | **Difference** | 3.20 | 0.00 | -1.50 | -10.60 | -0.80 | -0.10 | -0.40 | 0.10 | -0.20 | 0.00 |
| **12** | **Manual** | 0.20 | 2.20 | 0.20 | 0.20 | 0.10 | 0.20 | 2.20 | 0.40 | 0.40 | 0.10 |
|  | **Automated** | 0.20 | 2.70 | 0.20 | 0.20 | 0.10 | 0.20 | 2.20 | 0.40 | 0.50 | 0.10 |
|  | **Difference** | 0.00 | 0.50 | 0.00 | 0.00 | 0.00 | 0.00 | 0.00 | 0.00 | 0.10 | 0.00 |
| **13** | **Manual** | 38.70 | 38.70 | 7.10 | 18.30 | 9.20 | 38.70 | 17.30 | 0.70 | 0.40 | 4.90 |
|  | **Automated** | 38.40 | 40.00 | 6.20 | 14.20 | 10.10 | 39.30 | 17.20 | 0.80 | 0.40 | 4.90 |
|  | **Difference** | -0.30 | 1.30 | -0.90 | -4.10 | 0.90 | 0.60 | -0.10 | 0.10 | 0.00 | 0.00 |
| **14** | **Manual** | 57.20 | 57.30 | 11.80 | 56.20 | 22.90 | 57.00 | 32.40 | 43.40 | 16.90 | 0.90 |
|  | **Automated** | 57.10 | 57.20 | 10.90 | 55.60 | 18.60 | 57.10 | 31.40 | 48.70 | 16.80 | 1.10 |
|  | **Difference** | -0.10 | -0.10 | -0.90 | -0.60 | -4.30 | 0.10 | -1.00 | 5.30 | -0.10 | 0.20 |
| **15** | **Manual** | 50.50 | 57.90 | 26.20 | 49.60 | 8.30 | 9.10 | 53.30 | 57.10 | 57.60 | 1.00 |
|  | **Automated** | 57.10 | 58.40 | 12.60 | 49.20 | 2.90 | 7.60 | 51.90 | 57.20 | 57.70 | 1.00 |
|  | **Difference** | 6.60 | 0.50 | -13.60 | -0.40 | -5.40 | -1.50 | -1.40 | 0.10 | 0.10 | 0.00 |
| **16** | **Manual** | 52.60 | 57.10 | 42.30 | 51.50 | 16.00 | 15.30 | 56.60 | 56.60 | 56.50 | 1.20 |
|  | **Automated** | 56.10 | 57.10 | 29.90 | 52.40 | 10.20 | 14.30 | 56.60 | 56.60 | 56.40 | 1.50 |
|  | **Difference** | 3.50 | 0.00 | -12.40 | 0.90 | -5.80 | -1.00 | 0.00 | 0.00 | -0.10 | 0.30 |
| **17** | **Manual** | 21.50 | 57.70 | 5.30 | 10.00 | 0.80 | 1.60 | 56.80 | 57.50 | 56.50 | 0.40 |
|  | **Automated** | 34.10 | 57.60 | 3.70 | 5.30 | 1.20 | 1.40 | 56.90 | 57.60 | 56.30 | 0.50 |
|  | **Difference** | 12.60 | -0.10 | -1.60 | -4.70 | 0.40 | -0.20 | 0.10 | 0.10 | -0.20 | 0.10 |
| **CASE** | **Contour** | **WH** | **PC** | **RA** | **LA** | **RV** | **LV** | **AO** | **PA** | **SVC** | **IVC** |
| **18** | **Manual** | 55.60 | 56.60 | 41.60 | 48.70 | 5.40 | 8.20 | 55.60 | 56.20 | 56.00 | 1.20 |
|  | **Automated** | 56.50 | 56.60 | 31.00 | 45.70 | 7.10 | 8.90 | 55.20 | 56.30 | 56.00 | 1.20 |
|  | **Difference** | 0.90 | 0.00 | -10.60 | -3.00 | 1.70 | 0.70 | -0.40 | 0.10 | 0.00 | 0.00 |
| **19** | **Manual** | 45.80 | 56.90 | 1.70 | 43.00 | 1.80 | 4.30 | 56.40 | 56.50 | 20.10 | 0.50 |
|  | **Automated** | 52.10 | 57.00 | 1.70 | 36.90 | 1.80 | 4.30 | 56.40 | 56.40 | 20.20 | 0.40 |
|  | **Difference** | 6.30 | 0.10 | 0.00 | -6.10 | 0.00 | 0.00 | 0.00 | -0.10 | 0.10 | -0.10 |
| **20** | **Manual** | 56.00 | 56.00 | 16.60 | 55.90 | 18.20 | 53.70 | 56.60 | 47.60 | 16.30 | 9.20 |
|  | **Automated** | 56.00 | 56.10 | 16.90 | 56.00 | 16.60 | 53.90 | 56.60 | 56.00 | 15.90 | 9.10 |
|  | **Difference** | 0.00 | 0.10 | 0.30 | 0.10 | -1.60 | 0.20 | 0.00 | 8.40 | -0.40 | -0.10 |
| **Median Dmax (cc)** | **Manual** | 38.94 | 48.46 | 14.20 | 33.01 | 6.16 | 15.06 | 44.84 | 44.67 | 37.40 | 1.34 |
|  | **Automated** | 40.28 | 48.52 | 10.25 | 29.77 | 5.49 | 13.72 | 44.53 | 45.45 | 37.17 | 1.44 |
|  | **Per Substructure**  **Median Difference in Dmax**  (range) | 0.85  (-19.3–12.60) | 0.00  (-0.9–1.30) | -1.55  (-13.6–0.30) | -2.20  (-11.4–1.20) | 0.00  (-6.4–10.00) | 0.00  (-18.8–1.20) | -0.10  (-1.4–0.10) | 0.10  (-0.2–8.40) | -0.05  (-4.1–0.80) | 0.05  (-0.1–0.60) |
|  | **p value** | 0.6733 | 0.9413 | 0.3511 | 0.5603 | 0.9842 | 0.7429 | 0.7940 | 0.7941 | 0.8777 | 0.5408 |

**Supp. Table 10.** Cardiac substructure maximum dose (Gy) for manual and automated segmentation, with differences relative to manual.

*(PC = pericardium; WH = whole heart; RA = right atrium; RV = right ventricle; LA = left atrium; LV = left ventricle; PA = pulmonary artery; SVC = superior vena cava; IVC = inferior vena cava; AO = aorta)*

**Supp. Table 11**

|  | **Method** | **Model Building Scans** | | | | **Model Testing Scans** | | | | **Segmentation Time Per Patient (minutes)** | **Total Structures** | **Structures Included** | **Atlas for Manual** | **Notes** | **Open Source** |
| --- | --- | --- | --- | --- | --- | --- | --- | --- | --- | --- | --- | --- | --- | --- | --- |
|  |  | **Cancer Type** | **CT Type** | **IV**  **Contrast** | **Cases** | **Cancer Type** | **CT Type** | **IV**  **Contrast** | **Cases** |  |  |  |  |  |  |
| Garrett Fernandes 2021 (2) | DL | Lung | 3D | Some | 9 | Lung | 3D | Some | 50 | Not Specified | 9 | WH, LA, RA, LV, RV, AO, PA, SVC, IVC | Feng | n=99 clinical check  (fine-tuned version) | Access on request |
| Kaderka 2019 (3) | AB | Breast | 3D, DIBH | No | 6 | Breast | 3D, DIBH | No | 27 | 1–2 | 6 | WH, LA, RA, LV, RV, LAD | Feng | X | N |
| Spoor 2021 (4) | AB | Breast | 3D, DIBH | No | 30 | Breast | 3D, DIBH | No | 20 | Not Specified | 5 | WH, RA, LA, RV, LV | Feng | X | N |
| Maffei 2020 (5) | AB | Breast | 3D | No | 36 | Breast | 3D | No | 10 | 8 | 26 | WH, LA, RA, LV, RV, LVW (x5), AO, PA, SVC, IVC, CS, LMC, RCA (x4), LAD (x3), LCX (x2) | Duane | X | N |
| Haq 2019 (1) | DL | Lung | 3D | Some | 193 | Lung | 3D | Some | 48 | 0.2 | 12 | WH, PC, LA, RA, LV, RV, AO, PA, SVC, IVC, BA, BV | Feng | n=25 clinical check | Y |
| Van den Bogaard 2019 (6) | AB* | Breast | DIBH | No | 10 | Breast | 3D | No | 105 | 1 | 1 | LAD | Duane | X | N |
| Loap 2020 (7) | AB | Breast | 3D, DIBH | No | 20 | Breast | 3D, DIBH | No | 20 | 1 | 1 | LAD surrogate | Duane | X | N |
| Loap 2020 (8) | AB | Breast | 3D | No | 20 | Breast | 3D | No | 20 | 5 | 10 | WH, RA, LA, RV, LV, LVW (x5) | Duane | X | N |
| Jung 2019 (9) | AB | Not Specified** | 3D | Yes | 9 | Cross-validation | | | | <10 | 9 | WH, LC, RA, LV, RV, LMC, LAD, LCX, RCA | Feng | X | N |
| Luo 2019 (10) | AB | Not Specified | 3D | Not  Specified | 11 | Lung | 4D (Ave) | No | 49 | 10 | 11 | WH, RA, LA, RV, LV, AO, SVC, IVC, PA, PV, | Feng & Kong | n=20 clinical check | N |
| Morris 2020 (11)*** | AB | Breast | 3D,  4D (50%) | No | 13 | Breast | 3D,  4D (50%) | No | 11 | 1–10 | 13 | WH, RA, LA, RV, LV, AO, PA, SVC, IVC, PV, LMC, LAD, RCA | Feng | X | N |
| Zhou 2017 (12) | AB | “Thoracic malignancies” | DIBH, 4D (Ave) | Some | 14 | Lung | 4D (Ave) | No | 19 | 10 | 14 | WH, RA, LA, RV, LV, AO, PA, SVC, IVC, PV, LMC, LAD, LCX, RCA | Feng & Kong | X | N |
| Finnegan 2019 (13) | AB | Breast | 3D | No | 20 | Cross-validation | | | | 40 | 17 | LA, RA, RV, LV, LVBP, AO, PA, LAD, LMC, LCX, RCA, AV, MV, PVL, TV | Feng | X | Y |
| van Velzen 2021 (14) | DL | Not Specified | ECG-gated, dual-energy CTCA | Yes | 18 | Breast | 3D | No | 31 | Not Specified | 7 | RA, LA, RV, LVW (x1), LVBP, AO, PA | Not Specified | X | N |
| Farrugia 2021 (15) | AB | Lung | 4D (Average) | No | 20-40 | Lung | 4D (Ave) | No | 10 | Not Specified | 16 | WH, RA, LA, RA, LV, AO, PA, SVC, LMC, LAD, RCA, LCX, AV, MV, PVL, TV | Feng | X | N |

**Supp. Table 11.** A summary of the published cardiac substructure auto-segmentation tools (* = with additional processing steps; ** = chest abdo pelvis; *** = MR-fusion was also used for atlas building; **** = the original ABAS was run again twice including the amended outputs from 1^st^ (n=10) and 2^nd^ (n=10) runs).

*(DL = deep learning; ABAS = atlas-based; 3D = three-dimensional; 4D = four-dimensional; ECG = electrocardiogram; CTCA = CT coronary angiogram; PC = pericardium; WH = whole heart; RA = right atrium; RV = right ventricle; LA = left atrium; LV = left ventricle; PA = pulmonary artery; SVC = superior vena cava; IVC = inferior vena cava; AO = any portion of the aorta; CS = coronary sinus; LVBP = LV blood pool; BA = bilateral atria; BV = bilateral ventricles; LMC = left main stem coronary artery; LAD = left anterior descending coronary artery; LCX = left circumflex coronary artery; RCA = right coronary artery; PV = pulmonary vein; LVW = LV walls; PV = pulmonary valve*

**Supp. Table 12**

| **WH** | - SVC included in WH/PC in contrast scans (Feng suggests not to) |
| --- | --- |
| **PC** | - extends PC superiorly to cover entire aortic arch (not defined by Feng) |
| **RA** | - includes the majority of the auricle (not defined by Feng) |
| **RV** |  |
| **LA** | - includes the majority of the auricle (not defined by Feng) - variable lateral limit (not defined by Feng) |
| **LV** |  |
| **PA** | - encompasses the pulmonary trunk (not defined by Feng) - variable lateral limit for both pulmonary arteries (not defined by Feng) |
| **SVC** | - extends superiorly as where aorta begins to arch (not defined by Feng) |
| **IVC** | - variable inferior limit (not defined by Feng) |
| **AO** | - whole thoracic aorta included (not defined by Feng) |
| **ATRIA** | - variably similar to union structure of RA and LA (not defined by Feng) |
| **VENTRICLES** | - variably similar to union structure of RA and LA (not defined by Feng) |

**Supp. Table 12.** Notes on each cardiac substructure generated by the deep learning (where applicable).

*(PC = pericardium; WH = whole heart; RA = right atrium; RV = right ventricle; LA = left atrium; LV = left ventricle; PA = pulmonary artery; SVC = superior vena cava; IVC = inferior vena cava; AO = aorta)*

**REFERENCES**

1. Haq R, Hotca A, Apte A, Rimner A, Deasy JO, Thor M. Cardio-pulmonary substructure segmentation of radiotherapy computed tomography images using convolutional neural networks for clinical outcomes analysis. Phys Imaging Radiat Oncol. 2020;14(February):61–6.

2. Garrett Fernandes M, Bussink J, Stam B, Wijsman R, Schinagl DAX, Monshouwer R, et al. Deep learning model for automatic contouring of cardiovascular substructures on radiotherapy planning CT images: Dosimetric validation and reader study based clinical acceptability testing. Radiother Oncol. 2021;165:52–9.

3. Kaderka R, Gillespie EF, Mundt RC, Bryant AK, Sanudo-Thomas CB, Harrison AL, et al. Geometric and dosimetric evaluation of atlas based auto-segmentation of cardiac structures in breast cancer patients. Radiother Oncol. 2019;131:215–20.

4. Spoor DS, Sijtsema NM, van den Bogaard VAB, van der Schaaf A, Brouwer CL, Ta BDP, et al. Validation of separate multi-atlases for auto segmentation of cardiac substructures in CT-scans acquired in deep inspiration breath hold and free breathing. Radiother Oncol. 2021;163:46–54.

5. Maffei N, Fiorini L, Aluisio G, D’Angelo E, Ferrazza P, Vanoni V, et al. Hierarchical clustering applied to automatic atlas based segmentation of 25 cardiac sub-structures. Phys Medica. 2020;69(June 2019):70–80.

6. van den Bogaard VAB, van Dijk L V., Vliegenthart R, Sijtsema NM, Langendijk JA, Maduro JH, et al. Development and evaluation of an auto-segmentation tool for the left anterior descending coronary artery of breast cancer patients based on anatomical landmarks. Radiother Oncol. 2019;136:15–20.

7. Loap P, Tkatchenko N, Nicolas E, Fourquet A, Kirova Y. Optimization and auto-segmentation of a high risk cardiac zone for heart sparing in breast cancer radiotherapy. Radiother Oncol. 2020;153:146–54.

8. Loap P, Tkatchenko N, Kirova Y. Evaluation of a delineation software for cardiac atlas-based autosegmentation: An example of the use of artificial intelligence in modern radiotherapy. Cancer/Radiotherapie. 2020;24(8):826–33.

9. Jung JW, Lee C, Mosher EG, Mille MM, Yeom YS, Jones EC, et al. Automatic segmentation of cardiac structures for breast cancer radiotherapy. Phys Imaging Radiat Oncol. 2019;12(May):44–8.

10. Luo Y, Xu Y, Liao Z, Gomez D, Wang J, Jiang W, et al. Automatic segmentation of cardiac substructures from noncontrast CT images: accurate enough for dosimetric analysis? Acta Oncol (Madr). 2019;58(1):81–7.

11. Morris ED, Ghanem AI, Pantelic M V., Walker EM, Han X, Glide-Hurst CK. Cardiac Substructure Segmentation and Dosimetry Using a Novel Hybrid Magnetic Resonance and Computed Tomography Cardiac Atlas. Int J Radiat Oncol Biol Phys. 2019;103(4):985–93.

12. Zhou R, Liao Z, Pan T, Milgrom SA, Pinnix CC, Shi A, et al. Cardiac atlas development and validation for automatic segmentation of cardiac substructures. Radiother Oncol. 2017;122(1):66–71.

13. Finnegan R, Dowling J, Koh ES, Tang S, Otton J, Delaney G, et al. Feasibility of multi-atlas cardiac segmentation from thoracic planning CT in a probabilistic framework. Phys Med Biol. 2019;64(8).

14. van Velzen SGM, Bruns S, Wolterink JM, Leiner T, Viergever MA, Verkooijen HM, et al. AI-Based Quantification of Planned Radiation Therapy Dose to Cardiac Structures and Coronary Arteries in Patients With Breast Cancer. Int J Radiat Oncol Biol Phys. 2021;

15. Farrugia M, Yu H, Singh AK, Malhotra H. Autosegmentation of cardiac substructures in respiratory-gated, non-contrasted computed tomography images. World J Clin Oncol. 2021;12(2):95–102.
